# Single-Cell Lineage Tracing Uncovers Resistance Signatures and Sensitizing Strategies to FLT3 Inhibitors in Acute Myeloid Leukemia

**DOI:** 10.1158/0008-5472.CAN-24-3753

**Published:** 2026-04-02

**Authors:** Johanna Eriksson, Shuyu Zheng, Mihaela Popa, Jie Bao, Jun Dai, Wenyu Wang, Emmet McCormack, Anna Vähärautio, Jing Tang

**Affiliations:** 1 Research Program in Systems Oncology, Research Programs Unit, Faculty of Medicine, University of Helsinki, Helsinki, Finland; 2 Precision Oncology Research Group, University of Bergen, Bergen, Norway; 3 Kinn Therapeutics AS; 4 Department of Clinical Science, University of Bergen, Bergen, Norway; 5 Department of Hematology, Haukeland University Hospital, Bergen, Norway; 6 Centre for Pharmacy, Department of Clinical Science, University of Bergen, Bergen, Norway; 7 Centre for Cancer Biomarkers (CCBIO), University of Bergen, Bergen, Norway; 8 Foundation for the Finnish Cancer Institute, Finland

## Abstract

While FLT3 inhibitors have significantly improved the treatment of aggressive FLT3-mutated acute myeloid leukemia (AML), the emergence of resistance remains as a major challenge. Here, we applied our recently developed single-cell lineage tracing method ReSisTrace to identify cells that are intrinsically resistant or sensitive to the FLT3 inhibitors midostaurin and quizartinib in AML with FLT3-ITD mutations. Comparison of the gene expression profiles of these cells revealed transcriptional resistance signatures, including upregulation of GSPT1. Depletion of GSPT1 with CRISPR-Cas9-mediated knockout resulted in increased sensitivity of AML cells to quizartinib treatment. Further, targeting GSPT1 with the small molecule CC-90009 exhibited strong synergistic effects when combined with FLT3 inhibitors in the FLT3-ITD cell lines and primary AML patient samples. In addition, in an FLT3-ITD-positive AML patient-derived xenograft (PDX) mouse model, the CC-90009 and quizartinib combination showed significantly higher anti-tumor efficacy and prolonged overall survival compared to either treatment alone. Furthermore, compounds that induced transcriptomic changes opposite to the resistance signatures prompted cells to acquire FLT3 inhibitor-sensitive states. Vistusertib (mTOR inhibitor), linsitinib (IGF1R and insulin receptor inhibitor), and meisoindigo (IGF1R and Src family kinase inhibitor), all inhibiting pathways parallel to or downstream of oncogenic FLT3 signaling, were predicted and validated to sensitize FLT3-mutated cell lines and primary cells to FLT3 inhibitors. Collectively, these findings demonstrate the ability of ReSisTrace to unveil pre-existing transcriptional features of treatment vulnerability in hematological cancers and elucidate strategies for enhancing FLT3 inhibitor treatment efficacy in FLT3-ITD-mutated AML.

## Introduction

Acute myeloid leukemia (AML) is a highly aggressive and heterogeneous hematological malignancy marked by the infiltration of blood, bone marrow, and tissues by proliferative and abnormally differentiated cells of the myeloid lineage (1). The FMS-like tyrosine kinase-3 (FLT3) is a type 3 receptor tyrosine kinase crucial for the expansion of normal hematopoietic stem cells and it plays a pivotal role in the proliferation and anti-apoptosis of most primary AML cells. FLT3 is activated by binding of the FLT3 ligand (FL), which results in a signaling cascade activating multiple downstream signaling pathways, including PI3K/protein kinase B (AKT) and mitogen-activated protein kinase (MAPK) pathways (2). FLT3 is also one of the most commonly mutated genes in AML, with activating internal tandem duplications (ITD) or point mutations in the tyrosine kinase domain (TKD) occurring in approximately 20% and 7% of AML patients, respectively (3,4). In contrast to wild-type FLT3 signaling, FLT3-ITD not only activates PI3K and MAPK signaling but also triggers STAT5 signaling (2). The presence of FLT3-ITD mutations also serves as a prognostic marker, indicating a shorter disease-free and overall survival (5).

Development of FLT3 inhibitors has changed the standard of care for FLT3-mutated AML. Midostaurin (6) and the recently approved quizartinib are the only FDA-approved FLT3 inhibitors as a first-line treatment in combination with chemotherapy for adult patients with newly-diagnosed FLT3-ITD-positive AML. Notably, quizartinib was also approved as maintenance monotherapy for up to 3 years in patients who do not undergo subsequent hematopoietic stem cell transplantation. In addition, gilterinib has been approved as a monotherapy for adults with relapsed or refractory AML with FLT3 mutation (7). Although FLT3 inhibitors have significantly improved the survival of FLT3-mutated AML patients, primary therapeutic resistance and short-lived responses remain still as a frequent problem.

Resistance to FLT3 inhibitors can arise from specific FLT3-TKD mutations or mutations in other oncogenes (8–11), resulting in activation of proliferation and pro-survival signaling pathways. Nonetheless, emerging evidence indicates that non-genetic factors, such as epigenetic, transcriptional, and metabolic states, also play a crucial role in resistance to chemotherapy and targeted therapies in AML (12,13). Recent studies propose that early resistance to cancer therapies might also be mediated by non-genetic mechanisms, particularly through drug-tolerant persister (DTP) states encoded by pre-existing intrinsic transcriptional heterogeneity (14–16). These DTP cells, closely resembling clinical measurable residual disease (MRD), can subsequently undergo further evolution to acquire diverse drug-resistance mechanisms, including mutations (17). Novel therapeutic strategies aimed at targeting drug-tolerant persister cells are crucial to prevent the development of drug resistance and achieve MRD-negative complete remission, thereby reducing relapse rates and prolonging survival. However, the mechanisms behind this early resistance to FLT3 inhibitors in AML patients remain poorly understood.

In this study, we employed our recently established lineage-tracing method ReSisTrace (18) to investigate the transcriptional states preceding resistance against FLT3 inhibitors midostaurin and quizartinib in an FLT3-ITD-positive MOLM-13 cell line. We show that targeting a pre-resistance signature gene G1 to S phase transition 1 (*GSPT1*) with a selective degrader (CC-90009) exhibits strong synergy when combined with FLT3 inhibitors in FLT3-ITD-mutated AML cell lines and primary patient samples. In vivo, CC-90009 and quizartinib combination also shows higher anti-tumor effect and prolongs survival in an FLT3-ITD-positive AML PDX mouse model compared to the single treatments. We further searched the L1000 database (19) for compounds inducing gene expression changes opposite to the pre-resistance signatures. Linsitinib (insulin receptor inhibitor), vistusertib (mTOR inhibitor), and meisoindigo (Src family kinase inhibitor), all targeting pathways downstream of or parallel to oncogenic FLT3 signaling, were predicted and validated to pre-sensitize FLT3-ITD-positive cell lines and primary patient samples to FLT3 inhibitors. In conclusion, our study demonstrates the effectiveness of ReSisTrace in uncovering targetable, pre-existing transcriptional features of treatment resistance in hematological cancers. Importantly, the approach allowed us to identify synergistic and pre-sensitizing drugs that have the potential to increase FLT3 inhibitor treatment efficacy in FLT3-ITD-positive AML by preventing emergence of treatment resistance.

## Materials and methods

### Cell culture

AML cell lines MOLM-13 (RRID:CVCL_2119; kind gift from prof. Caroline Heckman; authentication of the cell line was performed with the Promega GenePrint24 System) and MV4-11 (#CRL-9591, ATCC, RRID:CVCL_0064), as well as the bone marrow mesenchymal stromal cell line HS-5 (RRID:CVCL_3720; kind gift from prof. Caroline Heckman) were grown in RPMI-1640 (#31870025, Thermo Fisher Scientific, Waltham, MA, USA) supplemented with 10% heat-inactivated fetal bovine serum (FBS) (#10500064, Thermo Fisher Scientific), 1% penicillin-streptomycin (#15140122, Gibco), and 1% GlutaMAX (#35050038, Thermo Fisher Scientific). Conditioned medium used for culturing primary AML patient samples was collected from 60% to 80% confluent HS-5 cell cultures after 72 hours of incubation, cleared by centrifugation, filtered through a 0.22-µm filter, and stored at -80°C. Cell lines were tested regularly for mycoplasma contamination with Venor GeM Classic mycoplasma detection kit (#11-1025, Minerva Biolabs, Berlin, Germany).

### Patient sample processing

Fresh-frozen bone marrow mononuclear cells from two FLT3-ITD-mutated AML patients at diagnosis, pre-treatment phase were obtained from the Finnish Hematology Registry and Clinical Biobank (FHRB; https://www.fhrb.fi/) with appropriate ethical approval. Patient characteristics are described in Table S1. Samples were selected based on high blast cell percentage and a high variant allele frequency of the FLT3-ITD mutation. Following thawing, cells were cultured for 2 to 3 hours in a mixture consisting of 25% HS-5 stromal cell-derived conditioned medium and 75% RPMI-1640, supplemented with 10% FBS, 1% penicillin-streptomycin, and 1% GlutaMAX, before seeding for drug combination testing. Mononuclear Cell Medium (MCM, #C-28030, PromoCell, Heidelberg, Germany) was also evaluated as a culturing medium for the primary AML cells. Protocols for processing the samples were approved by the Ethics Committees of Helsinki University Central Hospital.

### Small molecules

CC-90009 (#HY-130800), ENOblock (#HY-15858), meisoindigo (#HY-13680), pralatrexate (#HY-10446), vistusertib (#HY-15247), quizartinib (#HY-13001) were purchased from MedChemExpress (Monmouth Junction, NJ, USA), and AZD5438 (#S2621), methotrexate (#S1210), midostaurin (#S8064), and OTS167 (#S7159) from Selleckchem (Houston, TX, USA). Linsitinib (#CT-O906-2) was from ChemieTek (Indianapolis, IN, USA).

### ReSisTrace experimental workflow

Lineage barcode library (pBA439_UMI20) was synthesized, and lentiviruses were packaged as previously described (18). Two million MOLM-13 cells were transduced with the lentivirus library to obtain 20% transduction efficiency. Cells were centrifuged with the lentiviral particles at 800 x g for 30 minutes and incubated at normal growth conditions o/n, after which cells were washed and grown for 3 days before adding 0.5 ug/ml puromycin (#sc-108071, Santa Cruz Biotechnology, Dallas, TX, USA) for 9 days to remove cells without barcodes. Barcoded cells (1.2 x10^6^) were synchronized by incubating with 2 mM thymidine (#T1895, Merck, Darmstadt, Germany) for 24 hours, after which the cells were released from the thymidine block by washing, and 20,000 cells/well in 50 ul of growth medium were seeded on round-bottom 96-well plates. After 24 hours of incubation, 4 of the replicate wells were used for cell counting to ensure that the cells have approximately doubled, and 3 of the replicate wells were used as samples for the subsequent experiment. Each of these 3 samples were resuspended, and half of each sample (~20,000 cells) was analyzed by scRNA-seq as pre-treatment samples, while 25 ul of growth medium was added to the remaining half of each sample, after which the cells were treated with midostaurin (100 nM), quizartinib (10 nM), or DMSO (0.05%, # 0219605525, MP Biomedicals, Santa Ana, CA, USA) for 72 hours. The drug concentrations were chosen to kill ~70% of cells compared to DMSO-treated cultures. After the treatment, cells were washed 4 times, each time by adding 200 ul of medium, centrifuging the plates 300 x g 5 min, and removing 200 ul of medium. Cells were then allowed to recover for 3 (midostaurin), 4 (quizartinib), and 7 days (DMSO/growth control), after which ~20,000 cells from each sample were analyzed by scRNA-seq as post-treatment samples.

### Single-cell RNA-sequencing library preparation

Single-cell RNA sequencing libraries were generated with 10x Genomics’ Chromium Next GEM Single Cell 3' Kit v3.1 (#PN-1000269, 10x Genomics, Pleasanton, CA, USA), Chromium Next GEM Chip G Single Cell Kit (#PN-1000127), and Dual Index Kit TT Set A (#PN-1000215). Libraries were quantified with Agilent TapeStation (High Sensitivity D5000 ScreenTape system, Agilent Technologies, Santa Clara, CA, USA) and qPCR (KAPA Library quantification kit KK4835, Roche, Basel, Switzerland), and sequenced with NovaSeq 6000 (Illumina, San Diego, CA, USA).

### Lineage label mapping and processing

The lineage barcode sequence (5’-CTGGGGCACAAGCTTAATTAAGAATTCANNNNTGNNNNACNNNNGANNNNGTNNNNCTAGGGCCTAGAGGGCCC GTTTAAAC-3’) was incorporated as an individual gene *pBA439_UMI_20* into the GDCh38.d1.vd1 human reference genome alongside GENCODE v25 (RRID:SCR_014966) annotation to build the reference for running the Cell Ranger (v 5.0.1, RRID:SCR_017344) (20) pipeline to perform alignment and UMI quantification using a customized reference.

From the “possorted_genome_bam.bam” file generated by the “cellranger count” command, we extracted lineage label sequences for each cell. Specifically, we considered sequences mapped to the *pBA439_UMI_20* gene and the sub-pattern “CANNNNTGNNNNACNNNNGANNNNGTNNNNCT.” To account for sequencing errors, we applied the directional network-based method from UMI-tools (v1.0.1, RRID:SCR_017048) (21) to correct sequences that differed by a single base from the representative sequence. Additionally, we filtered out non-unique lineage label sequences expressed by more than four cells in the pre-treatment samples. We categorized cells from each pre-treatment sample into two groups: 1) pre-resistant cells with lineage labels matching those of corresponding post-treatment samples, and 2) pre-sensitive cells assigned to lineages not detected after treatment.

### Preprocessing of the scRNA-seq data

We used the Seurat (v4.0.4, RRID:SCR_016341) (22) to perform the data quality control, normalization, top variable gene selection, scaling, dimensionality reduction, and differentially expressed gene (DEG) analysis. For each ReSisTrace sample, the genes expressed in less than three cells were removed. We filtered the cells based on the distribution of the UMI counts ([20000, 200000] for pre-treatment sample; [10000, 100000] for post-treatment sample), the number of genes ([2500, 12000] for pre-treatment sample; [2000, 10000] for post-treatment sample), and percentage of mitochondrial transcripts (less than 15%). We used the “NormalizeData” function (default parameter setting) to normalize gene counts.

For clustering purposes, we merged the pre-treatment samples, including lineages with only one cell. After normalization and finding the top 3,000 variable genes by using the “FindVariableFeatures” function (default parameter setting), we assigned each cell a cell-cycle score based on its expression of G2/M and S phase markers by using the “CellCycleScoring” function. Then, we used the “ScaleData” function to scale gene counts, regressing out multiple variables, including the cell cycle scores, number of UMIs per cell, and percentage of mitochondrial gene expression. We used 30 principal components for UMAP projection and clustering, with Louvain algorithm and resolution 0.5.

### Sister cell similarity and sister-concordant genes

We pooled four pre-treatment samples together and defined the cells with matching labels within each treatment group as sister cells. Lowly expressed genes whose total normalized expressions are less than 0.61 (10% quantile) were removed. The Euclidean distances from top 1000 variant genes were calculated to evaluate the similarity between 1499 sister cell pairs and 1.44 x 10^8^ random cell pairs. The similarities for all sister cell pairs and 100 000 randomly selected random cell pairs were visualized in Fig. S1A.

For extracting the sister concordant genes, we considered only the lineages with two sister cells and removed the sister cells with the Euclidean distance of their transcriptomes larger than 17.86 (90% quantile). The genes with log2FCs significantly lower in the sister cell pairs compared to random cell pairs were defined as the sister-concordant genes (two-tailed t-test with an FDR-adjusted *P*-value threshold of 0.05).

### Pre-resistance gene expression signatures

The “FindMarkers” function (test.use = “wilcox”, logfc.threshold = 0, min.pct = 0) from Seurat R package was used to determine the differentially expressed genes by Wilcoxon rank sum test between pre-resistant and pre-sensitive groups in each sample. To minimize experimental noise from cases where both sisters were sampled in the scRNA-seq analysis before the treatment, we performed the differential analysis only on the cells lacking sisters in the same pre-treatment samples.

### Pathway analysis

Gene expression signature gene lists were first ranked by adjusted p-value, then by non-adjusted p-value, and finally by log2FC. The ranked gene lists were subjected to Gene Set Enrichment Analysis (GSEA) using the classic enrichment statistic implemented in GSEA software (v4.3.2, University of California San Diego and Broad Institute, RRID:SCR_003199) to inspect the MSigDB (RRID:SCR_016863) (23,24) v2023.2.Hs KEGG_LEGACY and BIOCARTA gene sets.

### Predicting drugs to target primed resistance

Four variants of pre-resistant gene expression signatures for both treatments were generated, including: 1) pre-resistance signature log2FC, 2) pre-resistance signature log2FC (gene list filtered by the Wilcoxon rank sum test *P*-value <0.05), 3) contrast log2FC between treatment and non-treatment control (pre-resistance signature log2FC - pre-fitness signature log2FC), and 4) contrast log2FC between treatment and non-treatment control (gene list filtered by bootstrapping test *P*-value < 0.05). Only sister-concordant genes were included in the analysis. Connectivity score defined in the Connectivity Map project (25) was used to assess the similarity of these signatures and the drug-induced consensus gene expression signatures, constructed using LINCS L1000 Connectivity Map data (RRID:SCR_016204), following the procedures described in previous work (26,27). Function “connectivityScore” (method = “fgsea”, nperm = 1000) from PharmacoGx R package (v3.0.2) (28) was used to calculate the connectivity scores. Drugs were ranked by their connectivity scores and filtered by *P*-value threshold <0.05 in all four pre-resistance signature variants.

### Drug combination testing and synergy scoring

Cell lines (600 or 800 cells/well for MOLM-13 and MV4-11, respectively) and primary patient samples (7500 cells/well) were seeded in 384-well plates in 25 µl of respective culture media using BioTek MultiFlo FX RAD (5 µl cassette) (Biotek, Winooski, VT, USA). Indicated concentrations of small molecules were added for 24 hours, after which indicated concentrations of midostaurin or quizartinib were added and incubated for 72 hours. Concentration ranges for drugs were chosen to induce 10% to 50% inhibition as single agents. As positive (total killing) and negative (growth control) controls 100 µM benzethonium chloride (#0522166110, MP Biomedicals) and 0.2% DMSO were used, respectively. Cell viability was then determined using CellTiter-Glo 2.0 assay (#G9243, Promega, Madison, WI, USA) according to manufacturer’s protocol. Each concentration and combination was tested in triplicate, and experiments were performed at least three times for cell lines and once or twice for patient samples. Representative results are shown.

Dose-response matrices were analyzed with SynergyFinder+ (https://tangsoftwarelab.shinyapps.io/synergyfinder/) (R package v3.8.2, RRID:SCR_019318) (29) using four different reference models, HSA (Highest Single Agent), Bliss (Bliss independence), Loewe (Loewe additivity), and ZIP (Zero Interaction Potency) to evaluate synergy between the drugs. HSA synergy scores were visualized as 3-D landscapes over the dose matrices. Mean synergy scores ≥ 5 were considered as synergistic. Significance of the mean synergy scores was assessed by bootstrapping (10 iterations).

### Generation of GSPT1-knockout cells

Two guide RNAs (gRNAs) targeting GSPT1 were independently designed using the Benchling software (https://www.benchling.com/, RRID:SCR_013955): gRNA_GSPT1_1: 5’ AATCCCAAAACCTAAGTCTG 3’, targeting exon 4, and gRNA_GSPT1_2: 5’ GGTATCAGTCTCTACATCAT 3’, targeting exon 12. Both GSPT1-targeting and non-targeting control gRNA pairs (Ctl_22: 5’ GAGTGATGCTTAGACTCCGT 3’ and Ctl_23: 5’ TTCGCACGATTGCACCTTGG 3’; (30) were cloned into the Lenti-CRISPR-V2 (https://www.addgene.org/52961/, RRID:Addgene_52961) vector by the Genome Biology Unit supported by HiLIFE and the Faculty of Medicine, University of Helsinki, and Biocenter Finland. Lentivirus packaging was provided by the Biomedicum Virus Core (HelVi-BVC) supported by HiLIFE and the Faculty of Medicine, University of Helsinki, and Biocenter Finland. MOLM-13 cells were transduced with the lentiviruses by centrifugation at 800 x g for 2 hours, followed by overnight incubation under standard culture conditions. The following day, cells were washed and cultured for an additional 24 hours before puromycin selection. After five days of selection, pooled populations of GSPT1-knockout and control MOLM-13 cells (expressing Cas9 and non-targeting gRNAs) were seeded in 96-well plates at 8,000 cells per well in 100 µl of culture medium and treated with either DMSO or quizartinib at concentrations of 1, 2, or 2,5 nM quizartinib in five to six replicates. After 72 hours, viable cell numbers were quantified using the TC20 Automated cell Counter (#1450102, Bio-Rad). Statistical significance was determined using two-tailed t-tests, with Holm’s method applied to adjust for multiple comparisons. Adjusted *P-*values <0.05 were considered statistically significant.

### Western blotting

Cells were harvested by centrifugation, washed twice with cold PBS, and lysed with RIPA buffer (#89900, Thermo Fisher Scientific) containing 2x Pierce protease inhibitor mixture (#A32955, Thermo Fisher Scientific) and 1 mM EDTA on ice for 30 min. Lysates were sonicated and cleared by centrifugation. Protein concentration was determined with Pierce BCA protein assay kit (#23227, Thermo Fisher Scientific). Then, samples were boiled for 5 min in 1x Laemmli sample buffer and 5% 2-mercaptoethanol. Proteins were resolved on 4–20% Mini-PROTEAN® TGX™ precast protein gels (Bio-Rad, Hercules, CA, USA) and transferred to Immobilon-FL PVDF membranes (#IPFL00005, Merck). After blocking with 2% BSA, membranes were incubated with the primary antibodies for 2h at RT or overnight at +4°C. Rabbit polyclonal antibody against GSPT1 (#10763-1-AP, Proteintech, Rosemont, IL, USA, RRID:AB_2115506), and as a loading controls mouse monoclonal antibodies against alpha-tubulin (DM1A, #ab7291, Abcam, Cambridge, UK, RRID:AB_2241126), alpha-actin (JLA20, #CP01, Merck, RRID:AB_566293), and beta-actin (#66009-1-Ig, Proteintech, RRID:AB_2687938) were used as primary antibodies. Immunodetection was performed with Azure 500 imager (Azure Biosystems, Dublin, CA, USA) using IRDye secondary antibodies (LI-COR Biosciences, Lincoln, NE, USA). Protein band signals were quantified using ImageJ (1.53c, NIH, Bethesda, MD, USA, RRID:SCR_003070).

### In vivo testing of drug combination efficacy in an AML PDX mouse model

The protocol for animal studies was approved by The Norwegian Animal Research Authority and conducted according to The European Convention for the Protection of Vertebrates Used for Scientific Purposes, in an AAALAC accredited institution. The PDX model (AML-PDX1) was developed from peripheral blood from a 59-year-old female with FLT3-ITD-positive AML using a previously described protocol (31). Female NSG mice (*n* = 32, 6 to 10 weeks old, JAX™ mice strain #005557, Charles River Laboratories, Wilmington, MA, USA, RRID:IMSR_JAX:005557) were inoculated intravenously via tail-vein with 1x10^6^ AML-PDX1 cells per animal using a 30-gauge insulin needle. Cells were administered as a suspension in phosphate-buffered saline with a final volume of 120 μL per syringe. Seven days after transplantation, the mice were allocated to control (vehicle), quizartinib (10 mg/kg daily, in 20% Hydroxypropyl-Beta-Cyclodextrin in sterile water, per os), CC-90009 (5 mg/kg daily, in 5% N-methyl-2- pyrrolidone (NMP) / 45% PEG 400 / 50% sodium chloride, subcutaneously), and combination treatment groups (*n* = 8 mice per group; randomized by weight and bioluminescence intensity). CC-90009 treatment was started a day before quizartinib treatment, to pre-sensitize leukemic cells. Animals were treated for two weeks, continuously. Disease progression was monitored through weekly bioluminescence imaging starting from the baseline, prior to the initiation of treatment. Optical images were acquired using an IVIS Spectrum system (PerkinElmer-Revvity, Waltham, MA, USA). For imaging purposes, mice with engrafted cancer cells received a contrast reagent, D-Luciferin (150 mg/kg, 250 mg/mL), via intraperitoneal injection. Data was analysed using the Living Image 4.7.3. software (PerkinElmer-Revvity). At the end of the study, mice were sacrificed following institutional guidelines as defined by weight loss, ruffled fur, and hind limb paralysis. Statistical significance was determined using two-tailed t-tests, with Holm’s method applied to adjust for multiple comparisons. For comparing Kaplan Meier curves, pairwise log-rank tests (also known as pairwise comparisons of survival curves) were performed using the pairwise_survdiff() function from the survminer R package (v0.5.0, RRID:SCR_021094), with Holm’s method applied to adjust for multiple comparisons. Adjusted *P-*values <0.05 were considered statistically significant.

## Results

### Establishing primed resistance signatures using lineage tracing

**Fig. 1:**
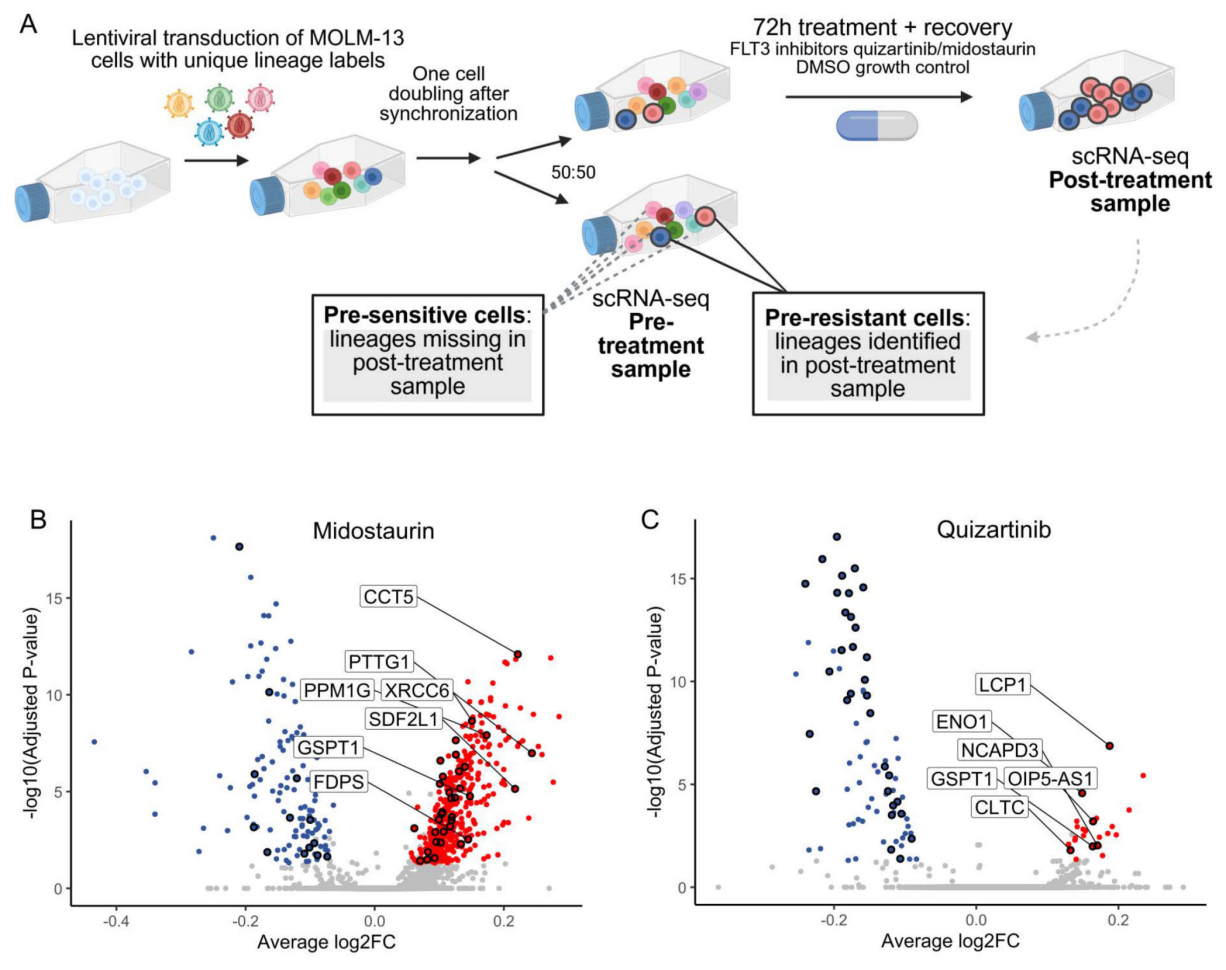
Utilization of ReSisTrace approach to study transcriptional states preceding resistance against FLT3 inhibitors in acute myeloid leukemia. A) Schematic representation of applying ReSisTrace approach to study primed resistance against FLT3 inhibitors in acute myeloid leukemia. Uniquely labeled, synchronized cells were permitted to duplicate once and were then divided into two groups for scRNA-sequencing (pre-treatment sample) and drug treatment. The lineage labels of cells that survived the treatment were determined by scRNA-sequencing (post-treatment sample) and were used to identify pre-resistant cells in the pre-treatment sample. Created in BioRender. Eriksson, J. (2005) https://BioRender.com/dd65wjz. (B-C) Midostaurin (B) and quizartinib (C) pre-resistance signatures were determined by comparing the gene expression profiles between the pre-resistant and pre-sensitive cells in the pre-treatment sample. Sister-concordant differentially expressed genes are shown in color. Genes differentially expressed specifically in the pre-resistance signatures compared to the pre-fitness signature obtained from the DMSO-treated growth control experiment are circled. Genes with the highest log2FC are labeled.

To elucidate transcriptional cell states that are primed to resist treatment with the FLT3 inhibitors quizartinib and midostaurin, we applied ReSisTrace in an FLT3-ITD-mutated AML cell line MOLM-13. In this approach, uniquely labeled and synchronized cells undergo one duplication before being randomly divided into two populations. One population is subjected to single-cell RNA-sequencing (scRNA-seq) analysis directly, while the other undergoes drug treatment (Fig. 1A). Cells subjected to drug treatment were exposed to either 100 nM midostaurin or 10 nM quizartinib for 72 hours to kill ~70% of the cells. Subsequently, the surviving cells were allowed to recover, and scRNAseq-analysis was performed to identify the drug-resistant lineages within the post-treatment samples. The lineage information was used to annotate corresponding sister cells in the pre-treatment sample as pre-resistant cells. Cell lineages present in the pre-treatment sample but absent in the post-treatment sample were annotated as pre-sensitive. We further analyzed a control sample treated with DMSO only, mimicking the growth conditions of the drug treatment experiments, to differentiate between transcriptional states associated with experiment-specific growth fitness and those specifically linked to primed resistance.

In pre- and post-treatment scRNA-seq samples, lineage labels consisting of 20 random bases were detected in approximately 85% of the cells passing quality control (Fig. S1B). On average, 71% of the lineages were unique for each treatment (Fig. S1C). In the pre-treatment samples, approximately 88% of the cells with lineage labels represented lineages with only one cell (Fig. S1D). We used only these cells in the downstream analyses to exclude lineages with increased basal growth rate and to reduce the rate for false-negative findings due to both sister cells ending up in the pre-treatment sample because of random sampling. We further validated that cells sharing the same lineage labels, i.e. sister cells, have significantly more similar transcriptomes than random cell pairs (Fig. S1A), suggesting that the sister cells can be used as proxies to study lineage-specific treatment resistance, in line with our results in the solid cancer setting (18).

We then explored the pre-treatment samples in the transcriptomic space. In the UMAP embedding, all cells within the pre-treatment samples were clustered together, indicating that the samples destined to undergo different treatments showed similar composition (Figs. S1E and S1F). Notably, no distinct subclones were found, indicating the homogeneity of the MOLM-13 cell line. Next, in the midostaurin pre-treatment sample, we determined 544 lineages as pre-resistant and 2015 lineages as pre-sensitive, based on the lineage labels observed or lacking in the post-treatment sample, respectively. Similarly, in the quizartinib pre-treatment sample, we annotated 344 lineages as pre-resistant and 3848 lineages as pre-sensitive. In the control pre-treatment sample, we found 801 lineages that remained in the respective post-treatment sample, and thus annotated as pre-fit cells, while 2840 lineages were annotated as pre-unfit cells. A UMAP projection did not reveal a clear separation between the pre-resistant and pre-sensitive cells, nor the pre-fit and pre-unfit cells (Figs. S1F-I), suggesting that there were no discernible subclonal patterns indicative of resistance.

**Fig. 2:**
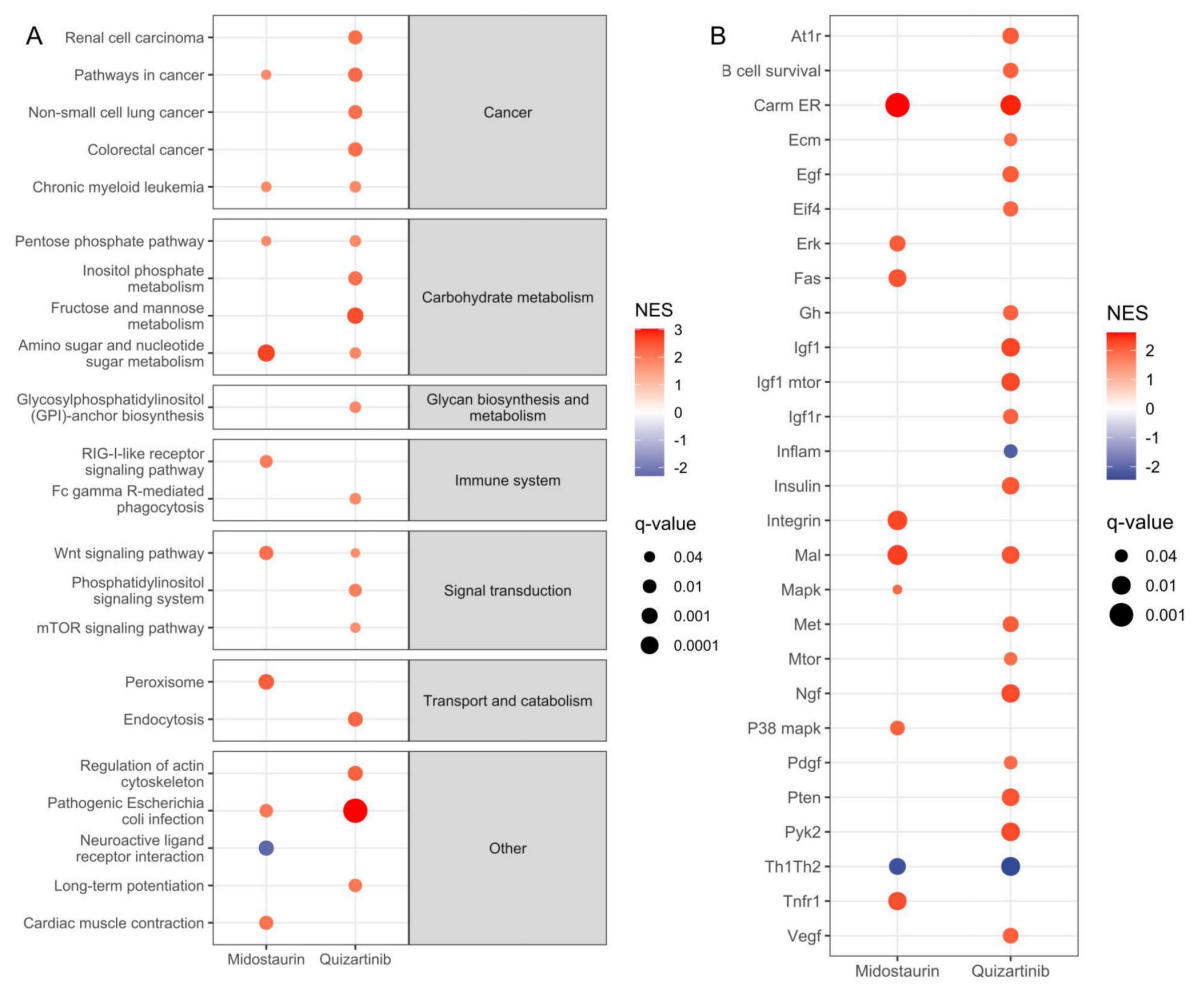
Gene set enrichment analysis. (A) KEGG and (B) BioCarta pathways specifically enriched among the midostaurin or quizartinib pre-resistance signature genes.

We then performed differential gene expression analysis between the pre-resistant and pre-sensitive cells using the non-parametric Wilcoxon rank sum test, to obtain the midostaurin and quizartinib pre-resistance signatures (Figs. 1B and 1C; full gene lists are shown in Tables S2 and S3). Additionally, we compared the expression profiles of the pre-fit and pre-unfit cells in the DMSO-treated growth control experiment to elucidate the pre-fitness signature (Table S4). To determine the potential pathways associated with primed FLT3 inhibitor resistance, we ran gene set enrichment analysis (GSEA) on the pre-resistance and pre-fitness signature gene lists, ranked by adjusted *P*-value, *P*-value, and log2 fold change (log2FC). We found that many KEGG pathways linked to cell cycle, DNA replication, and DNA repair were enriched among the genes up-regulated in both pre-resistant and pre-fit cells (full lists of significant KEGG pathways are shown in Table S5). Moreover, KEGG pathways related to carbohydrate metabolism, including fructose and mannose metabolism, inositol phosphate metabolism, pentose phosphate pathway, as well as amino sugar and nucleotide sugar metabolism, were enriched among genes specifically up-regulated in the pre-resistant cells (Fig. 2A). When comparing the midostaurin and quizartinib pre-resistance signatures, we found that the Wnt signaling pathway was up-regulated in both of them, consistent with previous findings that activation of Wnt/beta-catenin signaling decreases the potency of FLT3 inhibitors in FLT3-ITD-mutated cells (32). In contrast, phosphatidylinositol signaling system and mTOR signaling pathway were up-regulated only in the quizartinib pre-resistance signature. The analysis of the BioCarta pathways also indicated the up-regulation of mTOR signaling in quizartinib pre-resistance signature, while MAPK and ERK signaling pathways were up-regulated in the midostaurin pre-resistance signature (Fig. 2B; full lists of significant BioCarta pathways are shown in Table S6).

### Targeting individual pre-resistance signature genes with inhibitors

As we observed an overlap between the top up-regulated genes in the pre-resistance signatures and those in the growth control (pre-fit versus pre-unfit cells), we sought to identify genes exhibiting increased expression specifically within the pre-resistance signatures compared to the pre-fitness signature. To accomplish this, we employed permutation and bootstrapping tests to assess the significance of the fold change differences between the signatures. Our analysis revealed that 41 genes were significantly (adjusted *P*-value < 0.05 in pre-resistant vs pre-sensitive) and specifically (permutation and bootstrapping test between pre-resistance and pre-fitness log2FC, *P* < 0.05) up-regulated in midostaurin pre-resistant cells, and 7 genes in quizartinib pre-resistant cells when compared to both their respective pre-sensitive cells and the pre-fit signal within the DMSO control (Figs. 1B and 1C; Tables S7 and S8; full results are shown in Tables S2 and S3).

**Fig. 3:**
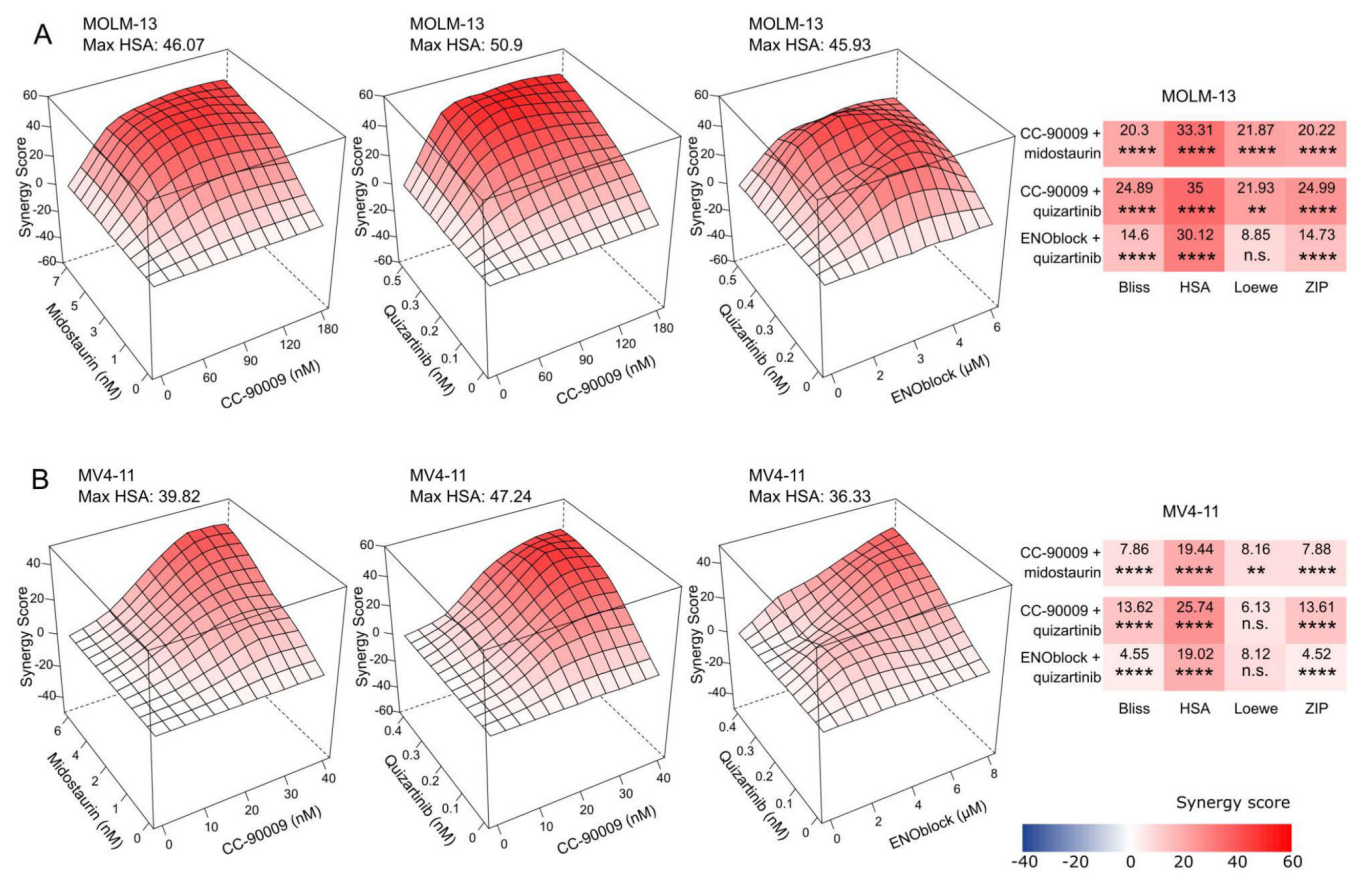
Synergistic effect of drugs targeting individual pre-resistance genes in combination with FLT3 inhibitors midostaurin or quizartinib. HSA synergy landscapes and mean synergy scores for CC-90009 and ENOblock in combination with midostaurin or quizartinib in MOLM-13 (A) and MV4-11 cells (B). *P*-values were determined by bootstrapping test; n.s. = not significant, *P < 0.05, **P < 0.01, ***P < 0.001, ****P < 0.0001.

We selected one gene, G1 to S phase transition 1 (*GSPT1*), which was the only targetable gene present among the top genes in the pre-resistance signatures for both midostaurin and quizartinib treatments (Tables S7 and S8), to investigate whether combining a GSPT1 inhibitor with FLT3 inhibitors could enhance treatment efficacy. *GSPT1* encodes a protein involved in translation termination, and it could be targeted for selective ubiquitin-mediated degradation with a molecular glue CC-90009 (33). Our experimental approach involved pre-treating MOLM-13 cells with CC-90009 for 24 hours, followed by the addition of midostaurin or quizartinib for an additional 72 hours. CC-90009 showed synergy with both midostaurin and quizartinib according to the common synergy scoring methods, including Bliss (Bliss independence), HSA (Highest Single Agent), Loewe (Loewe additivity), and ZIP (Zero Interaction Potency) (Fig. 3A; dose-response matrices in Fig. S2A). Importantly, the combination was synergistic also in another FLT3-ITD-positive cell line, MV4-11 (Figs. 3B and S2B). To confirm the expected mechanism, we verified by western blotting that CC-90009 treatment led to the degradation of GSPT1 in both MOLM-13 and MV4-11 cells (Fig. S3). In MOLM-13, we observed predominantly low expression of the long isoform (~80 kDa) and elevated levels of a shorter (~40 kDa) isoform/fragment. Following CC-90009 treatment, there was a 73% to 80% reduction in the shorter isoform/fragment compared to the DMSO-treated control (Fig. S3A). In turn, MV4-11 cells exhibited elevated expression of the long GSPT1 isoform, accompanied by the presence of the shorter fragment. Both isoforms were significantly reduced by 39% to 58% after CC-90009 treatment (Fig. S3B). To further investigate the role of GSPT1 in primed resistance to FLT3 inhibitors, we used a CRISPR-Cas9 system with double guide RNAs (gRNAs) to knockout GSPT1 in MOLM-13 cells. To minimize confounding effects of clonal variation, we analyzed pooled populations of GSPT1-knockout (KO) cells and non-targeting double gRNA-expressing control cells. Knockout led to a 65% reduction in GSPT1 protein levels (Fig. S4A). According to the DepMap Chronos data (https://depmap.org/portal/), GSPT1 depletion has a strong negative fitness effect in MOLM-13 cells (Chronos gene effect -1.59), which likely contributed to the difficulty in generating stable high-efficiency GSPT1-KO pools. Consistent with this, GSPT1-KO cells exhibited a transient reduction in growth rate compared to controls (Fig. S4B), lasting approximately for one week post knockout. This suggests the cells may undergo adaptation in response to GSPT1 loss. When treated with quizartinib for 72 h—one week after transduction—GSPT1-KO cells showed a dose-dependent increase in sensitivity to the drug when compared to the control cells (Fig. S4C). Taken together, these results support a functional role for GSPT1 in mediating primed resistance to FLT3 inhibitors and suggest that GSPT1 degraders may enhance the efficacy of FLT3 inhibitor therapy in FLT3-ITD-mutated AML.

In our search for specific combination therapies for quizartinib, we further selected a top gene enolase 1 (*ENO1*) from the pre-resistance signature (Table S8). *ENO1* is a gene encoding a glycolytic enzyme alpha-enolase, which can be targeted by ENOblock (34). Again, we pre-treated the cells with ENOblock before adding quizartinib. In both MOLM-13 and MV4-11 cell lines, ENOblock showed synergy with quizartinib according to Bliss, HSA, and ZIP scores (Fig. 3; dose-response matrices in Fig. S2), suggesting that ENO1 may play a role in primed resistance against FLT3 inhibitors in FLT3-ITD-mutated AML.

### Targeting pre-resistant states by drugs with opposite gene expression signatures

**Fig. 4:**
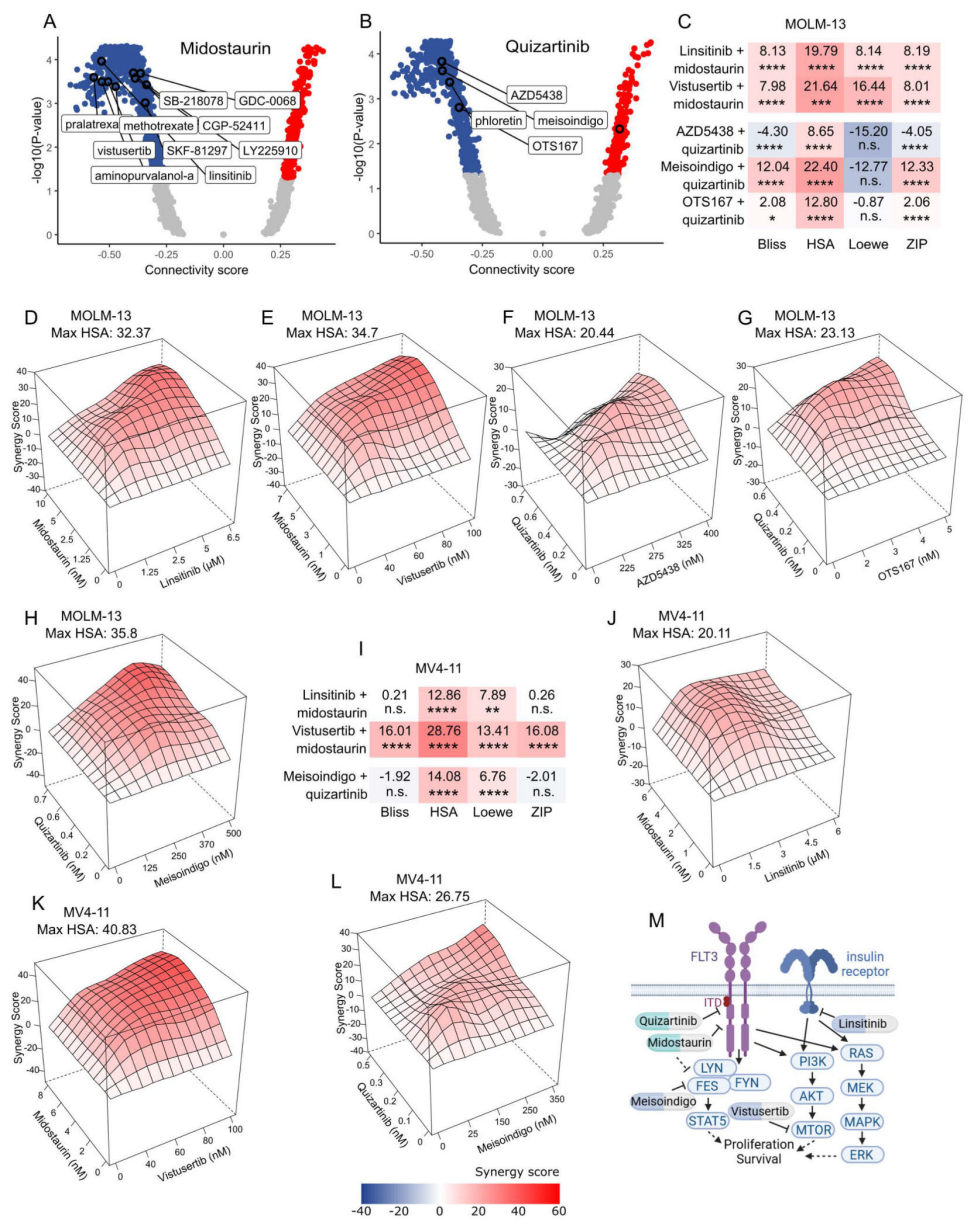
Targeting pre-resistant states with drugs with opposite gene expression signatures. (A-B) Connectivity scores between the L1000 drug perturbed signatures and unfiltered midostaurin (A) and quizartinib (B) pre-resistance signatures. Drugs with significant connectivity scores in all signature variants are circled. (C-L) Mean synergy scores (C and I) and HSA synergy landscapes (D-H, J-L) for the indicated drugs in combination with midostaurin or quizartinib in MOLM-13 (C-H) and MV4-11 (I-L) cells. (C and I) *P-*values were determined by bootstrapping test; n.s. = not significant, *P < 0.05, **P < 0.01, ***P < 0.001, ****P < 0.0001. (M) Schematic representation of pathways targeted by the top-performing pre-sensitizing drugs. Created in BioRender. Eriksson, J. (2025) https://BioRender.com/ojbcwv7.

In addition to targeting individual genes in the pre-resistance signature, we employed a systematic strategy to target primed resistance in a more comprehensive manner. This involved the use of small molecules that would shift cancer cells toward the pre-sensitive states, thereby enhancing their responsiveness to FLT3 inhibitor treatment. To this end, we leveraged the pre-resistance signatures obtained from the ReSisTrace analysis to predict such compounds. We queried the L1000 database (19) for drugs that would induce gene expression changes opposite to the pre-resistance signatures (i.e. similar to the pre-sensitivity signatures). To obtain more robust results, we used multiple variants of the pre-resistance signatures, two of which were adjusted for gene expression patterns associated with experimental growth fitness (see Methods). The drugs were filtered by negative connectivity scores with *P*-value < 0.05 across all the pre-resistance signature variants (Figs. 4A and 4B; Table S9). Notably, some of the identified drugs target kinases downstream of FLT3, such as vistusertib, which inhibits mTOR, GDC-0068 inhibiting AKT, and meisoindigo inhibiting multiple SRC family kinases along with IGF1R. Additionally, some of the identified drugs inhibit kinases activating the same signaling pathways as FLT3, such as CGP-52411 (EGFR inhibitor) and linsitinib (IGF1R and insulin receptor inhibitor). According to our scRNA-seq data and the DepMap Portal (https://depmap.org/), only the insulin receptor is expressed in MOLM-13 cells, whereas IGF1R and EGFR are not. Among the other potential pre-sensitizing drugs were CDK inhibitors, a CHEK1 inhibitor, and OTS167, which targets MELK and has been reported to inhibit FLT3 protein translation and synergize with the FLT3 inhibitor gilterinib in FLT3-ITD-mutated AML (35). When combined with midostaurin, linsitinib (insulin receptor inhibitor) and vistusertib (mTOR inhibitor) demonstrated synergism across all four synergy scoring methods (Figs. 4C-4E; Dose-response matrices in Fig. S2A). In turn, in combination with quizartinib, AZD5438 (CDK inhibitor) displayed synergism only according to the HSA scores (Figs. 4C, 4F, and S2A), while OTS167 (MELK inhibitor) exhibited additive effect or low synergism according to three synergy scoring methods (Figs. 4C, 4G, and S2A). Notably, meisoindigo (SRC family kinase and IGF1R inhibitor) demonstrated higher synergism with quizartinib according to Bliss, HSA, and ZIP methods (Figs. 4C, 4H, and S2A). We then evaluated the effect of the combinations, which showed the highest synergism, in the other FLT3-ITD-positive cell line, MV4-11. We found that vistusertib consistently displayed the highest synergism with midostaurin, while linsitinib with midostaurin and meisoindigo with quizartinib demonstrated synergism according to HSA and Loewe methods (Figs. 4I-4L and S2B). Interestingly, all the top-performing predicted drugs, linsitinib, vistusertib and meisoindigo, inhibit pathways downstream or parallel to FLT3 signaling (Fig. 4M). Our results indicate that these pathways are activated in the pre-resistant cells and inhibiting these pathways pre-sensitize FLT3-ITD-mutated cells to FLT3 inhibitors.

### Validating drug combinations in FLT3-ITD-mutated patient samples

**Fig. 5:**
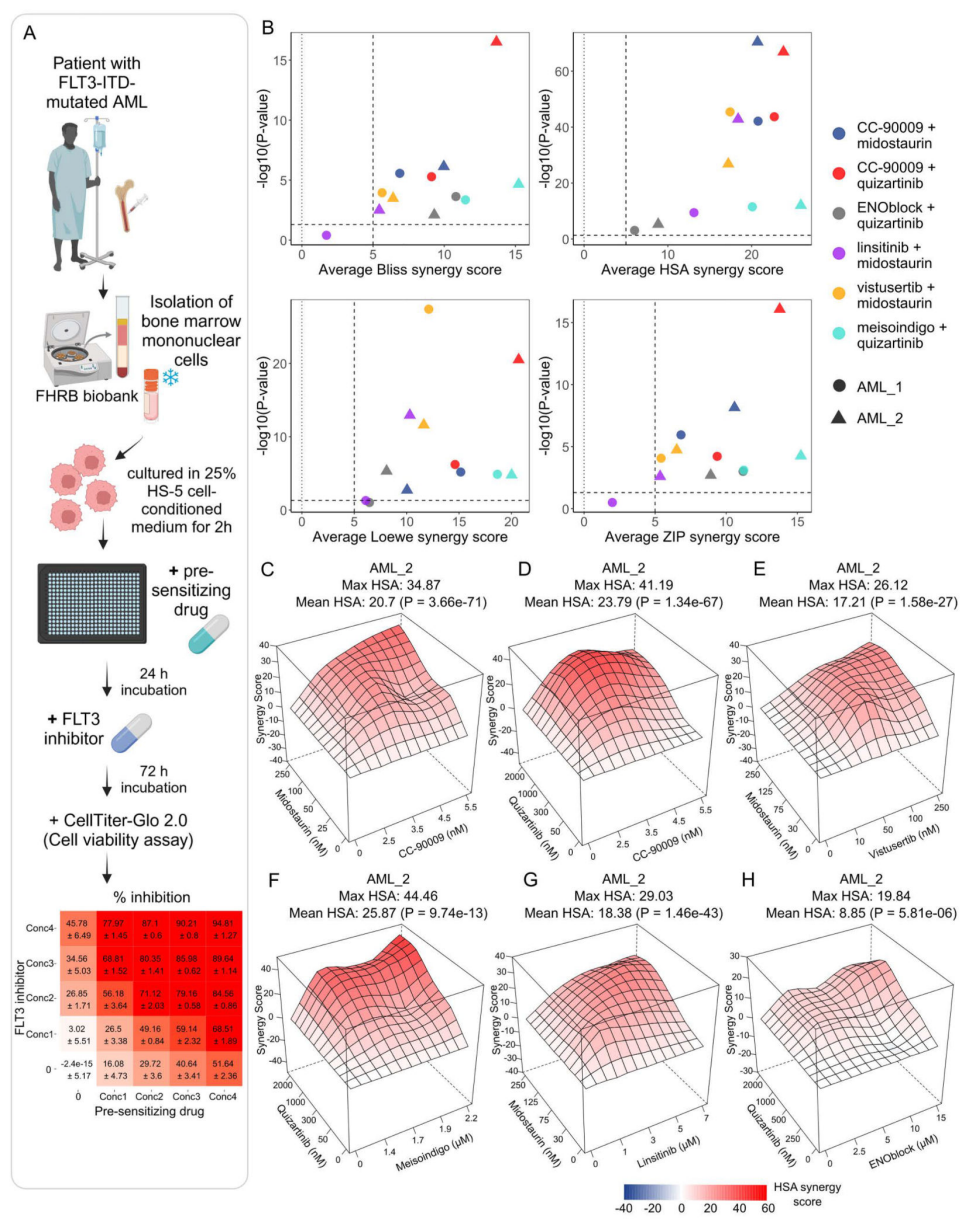
Validation of drug combinations in patient samples. (A) Schematic representation of testing drug combinations in primary AML patient samples. Created partly in BioRender. Eriksson, J. (2025) https://BioRender.com/xqrxl84. (B) Average synergy scores according to different synergy scoring methods for the six drug combinations in two FLT3-ITD-mutated AML patient samples. (C-H) HSA synergy landscapes for the six drug combinations in patient sample AML_2. *P*-values were determined by bootstrapping test.

We then evaluated the efficacy of the drug combinations in two diagnosis-stage FLT3-ITD-mutated AML patient samples (Fig. 5A), with blast cell percentage ≥ 58% and an FLT3-ITD variant allele frequency ≥ 70% (Table S1). Initially, we cultured the samples in both Mononuclear Cell Medium (MCM) and 25% HS-5 stromal cell-conditioned RPMI-1640 medium. Despite previous reports showing that HS-5 cell-conditioned medium protects FLT3-ITD-mutated primary AML cells from FLT3 inhibitors (36), we chose this medium for our experiments as it more closely mimics the in vivo environment and supported the growth of the primary cells. MCM, in contrast, did not support primary cell growth for the required 4 days (Fig. S5A).

In single-drug treatments, midostaurin and meisoindigo were less effective in primary AML samples compared to MOLM-13 and MV4-11 cell lines. Interestingly, primary cells were more sensitive to CC-90009 than the cell lines (Figs. S2 and S5). Quizartinib did not achieve 50% inhibition in the primary AML cells even with the highest concentration tested (2 µM), while ENOblock was either ineffective or promoted growth at concentrations up to 15 µM (Fig. S5). Among the drug combinations, CC-90009 consistently exhibited high synergy with quizartinib and midostaurin in both samples, as confirmed by all four synergy scoring methods (Figs. 5B-5D, S6A, S6B; dose-response matrices in Fig. S5). Vistusertib combined with midostaurin, and meisoindigo with quizartinib showed synergism in both samples according to all synergy scores (Figs. 5B, 5E, 5F, S6C, S6D; dose response Figs. S5). Linsitinib and midostaurin combination displayed significant synergy according to all synergy scoring methods but only in sample AML_2 (Figs. 5B, 5G, S6E; dose response Fig. S5) suggesting that certain resistance-conferring pathways may be activated in a subset of patients. Meanwhile, ENOblock combined with quizartinib showed synergy in both samples (Figs. 5B, 5H, and S6F) but the overall inhibition levels remained low, likely due to the ineffectiveness of both drugs as single agents (Fig. S5).

### Validating the synergistic effect of CC-90009 and quizartinib in an AML PDX mouse model

**Fig. 6:**
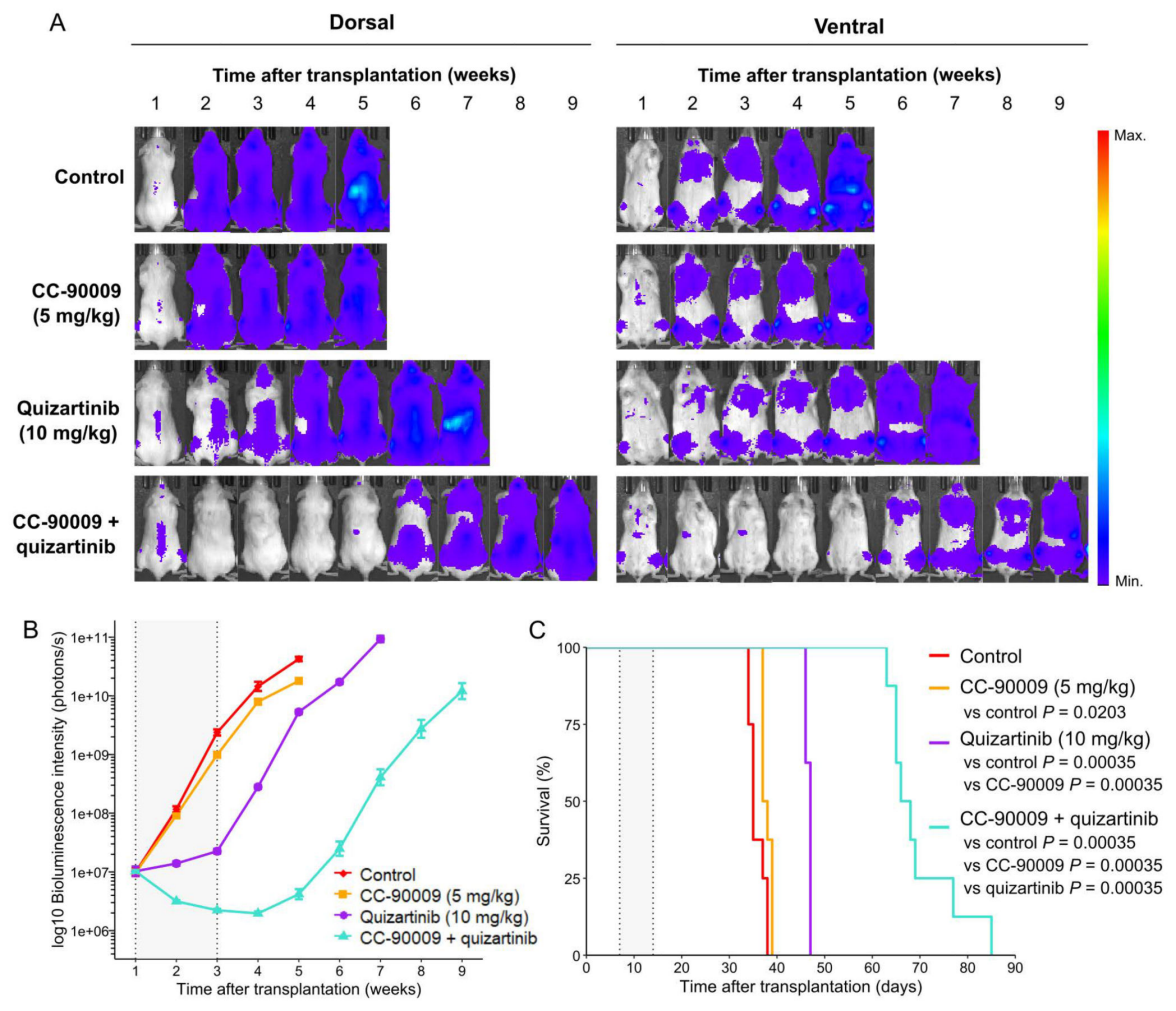
Efficacy of CC-90009 and quizartinib in an FLT3-ITD-positive AML PDX mouse model. (A-B) Representative bioluminescent images (A) and bioluminescence quantification (mean ± standard error of mean) (B) of AML-PDX1-engrafted mice at indicated timepoints after transplantation. Mice (*n* = 8 per group) were treated daily with the indicated therapies for two weeks starting one week after transplantation (grey area). (C) Kaplan-Meier survival curves of the AML-PDX1-engrafted mice. Adjusted *P-*values were calculated using pairwise log-rank tests.

To evaluate the in vivo efficacy of CC-90009 and quizartinib—the combination demonstrating the highest synergy in ex vivo cultures of AML patient samples—we utilized a pre-established luciferase-expressing FLT3-ITD-positive patient-derived xenograft (AML-PDX1) mouse model. AML-PDX1 cells were intravenously injected into NSG mice, and seven days post engraftment, the animals were randomized by weight into four treatment groups: vehicle, quizartinib (10 mg/kg), CC-90009 (5 mg/kg), or the combination. Tumor burden was assessed through bioluminescence imaging, and mice were monitored for weight loss and overall survival until they were euthanized due to symptomatic disease progression. Treatments were administered daily for 14 days, with CC-90009 initiated one day prior to quizartinib to pre-sensitize leukemic cells. The treatments were generally well tolerated, with no significant weight loss observed throughout the study (Fig. S7). Apart from transient ruffled fur, no other treatment-related clinical signs were noted. The weight loss observed in all groups during the later stages of the experiment was attributed to advanced leukemic progression rather than treatment-related toxicity. CC-90009 monotherapy showed low antitumor activity, while quizartinib monotherapy displayed stable disease throughout the treatment phase (Fig. 6). However, this effect was not sustained after treatment cessation, indicating limited durability of the single-agent FLT3 inhibition. Importantly, the combination therapy significantly reduced tumor burden during treatment, demonstrated a sustained anti-leukemic effect post-treatment, and improved the overall survival compared to all other groups (Figs. 6 and S7).

Taken together, targeting GSPT1 with a molecular glue degrader exhibits strong synergy with FLT3 inhibitors in FLT3-ITD-positive AML both in vitro and in vivo and represents a promising approach to enhance treatment efficacy in FLT3-ITD-mutated AML. Furthermore, the three top-performing drugs, vistusertib, linsitinib and meisoindigo, predicted to pre-sensitize cells to FLT3 inhibitors, demonstrated synergy with the FLT3 inhibitors in both FLT3-ITD mutated patient samples and cell lines. These findings validate the effectiveness of our ReSisTrace lineage-tracing method for identifying new, rational drug combinations targeting primed treatment resistance in hematological cancers.

## Discussion

Despite considerable progress in developing more potent and selective FLT3 inhibitors, the emergence of resistance persists as a significant challenge. Studies investigating AML patient relapses after FLT3 inhibitor therapy have consistently identified alterations in genes within the RAS/MAPK pathway, particularly NRAS and KRAS, as the most prevalent genetic aberrations associated with acquired resistance, alongside novel FLT3 mutations (9–11). Moreover, FLT3-independent activation of RAS/MAPK and/or PI3K/AKT/mTOR signaling pathways, along with the sustained expression of genes involved in FLT3-mediated cellular transformation, has been observed in FLT3 inhibitor-resistant cell lines and primary samples (37,38). Additionally, non-genetic mechanisms, such as soluble factors from the bone marrow microenvironment, can activate the downstream RAS/MAPK, PI3K/AKT, and mTOR signaling in AML cells, contributing to early treatment resistance against FLT3 inhibitors (39–41). Furthermore, intrinsic transcriptional heterogeneity can also induce drug tolerance in cancer cells (14). Our ReSisTrace lineage-tracing method offers a means to unbiasedly detect pre-existing transcriptional states leading to resistant fates. Short-term drug treatment is used to identify transcriptional states associated with early intrinsic drug-tolerance present prior to the treatment, without capturing possible stable resistance clones harboring resistance-conferring mutations that outgrow during prolonged drug exposure (39). Applying the method on FLT3-ITD-positive cells unveiled transcriptional states characterized by increased signaling of the aforementioned resistance-conferring pathways, priming cells for resistance against FLT3 inhibitors within the treatment-naïve cell population. Reverting these cell states that are predisposed to survive the treatment, already before more stable resistance emerges, could represent a viable strategy to enhance long-term remission rates in FLT3-ITD-positive AML patients.

Our lineage-tracing method not only allows characterization of resistant populations prior to the treatment but also reveals the vulnerable states that could be induced via pre-treatments. We targeted primed resistance with drugs that were predicted to pre-sensitize cells to FLT3 inhibitors based on the similarity between our pre-sensitivity signature and the L1000 drug-perturbed consensus signatures. Among these drugs, many target pathways that are parallel to or downstream of oncogenic FLT3 signaling (Fig. 4M). For instance, vistusertib inhibits mTOR activity, attenuating any FLT3-ITD-independent resistance-promoting mTOR signaling when combined with an FLT3 inhibitor. Consistent with this, a dual PI3K/mTOR inhibitor PF-04691502 has been found to display synergistic cytotoxicity with quizartinib in FLT3-ITD AML cells (42). Another predicted drug, linsitinib, selectively targets the insulin receptor and IGF1R (43). Notably, only the insulin receptor is moderately expressed in the MOLM-13 and MV4-11 cell lines, according to our scRNA-seq data and the DepMap Portal (https://depmap.org/portal), suggesting that linsitinib elicits its effect through inhibiting insulin receptor and not IGF1R. Upon binding of insulin or IGF1, INSR activates multiple downstream pathways, including PI3K/AKT/mTOR and RAS/MAPK/ERK signaling (44). IGF1R is also targeted by meisoindigo, but since IGF1R is not expressed in the MOLM-13 and MV4-11 cells, meisoindigo’s main mechanism of action in these cell lines likely involves inhibition of several non-receptor tyrosine kinases, including FES, FER, and the SRC family kinases LYN, FYN, YES1, LCK, and SRC (45). Among these, LYN and FES are expressed in MOLM-13 and MV4-11 and play key roles in oncogenic FLT3 downstream signaling (46,47). In primary AML cells, meisoindigo demonstrated strong synergy with quizartinib in both samples, with synergy scores even higher than in the cell lines. Interestingly, meisoindigo has been used to treat chronic myeloid leukemia in China for decades (48) and has also been reported to induce apoptosis in AML cell lines and primary AML cells (49). Notably, vistusertib and linsitinib, when combined with midostaurin, also showed enhanced synergy in the two samples. Together, these findings suggest that the resistance-conferring pathways targeted by these pre-sensitizing drugs may play an important role in at least a subset of FLT3-ITD-mutated AML patients. Further studies are needed to elucidate the mechanisms driving the activation of these pathways and the precise actions of these drug combinations, as well as their effects on normal hematopoietic and other healthy cells. In phase I trials, vistusertib and linsitinib were generally well tolerated, with dose-limiting toxicities of fatigue and mucositis (vistusertib) (50) and hyperglycemia, vomiting, fatigue, and QTc prolongation (linsitinib) (51). In a phase III trial, meisoindigo was also well tolerated in chronic myeloid leukemia, with bone, joint, or muscle pain as the main side effects and no cases of severe myelosuppression (52).

Among the drugs predicted to pre-sensitize cells to FLT3 inhibitors, some showed low synergy or even antagonistic effects when tested in MOLM-13 in combination with FLT3 inhibitors. This discrepancy might stem from decreased accuracy of predictions when comparing our pre-resistance signatures against the L1000 phase 1 consensus signatures, generated from drug-perturbed gene expression profiles of cell lines derived from solid cancers, not including data from blood cancer cell lines. Furthermore, the detection of subtle transcriptional changes that mediate primed resistance against FLT3 inhibitors, while considering the accompanying growth fitness-related alterations, presents several challenges. The up-regulation of pathways downstream of oncogenic FLT3 signaling could 1) confer resistance to the FLT3 inhibitors, indicated by our GSEA findings and pathways targeted by the validated pre-sensitizing drugs, and 2) promote increased proliferation and fitness in the non-treatment control, given that FLT3-ITD acts as the oncogenic driver of MOLM-13 cells. This dual effect complicates the distinction between resistance-related alterations and those providing growth advantage. Additionally, cell cycle synchronization via thymidine block introduces stress and imposes a non-random selection pressure to all samples, potentially inducing noise that obscures the pre-resistance signal, and thus warrants careful consideration when analyzing sensitive cancer models. As a further note, technical factors contribute to underestimating the number of true pre-sensitive and pre-unfit cell lineages. These include 1) random loss of lineages during sample splitting—when dividing cells into two samples for sequencing and drug treatment, both sister cells from some lineages may end up in the same sample, 2) cell loss of during scRNA-seq preparation, and 3) partial sampling of the recovered post-treatment sample. Consequently, the pre-sensitive and pre-unfit categories likely include false negatives, which dilute the differential gene expression signal between groups. Importantly, however, all identified pre-resistant and pre-fit lineages represent true positives.

As a model for FLT3-ITD-mutated AML, MOLM-13 cell line is heterozygous for the FLT3-ITD mutation, also expressing the wild-type FLT3 receptor. It has been shown that the ligand-dependent activation of wild-type FLT3 is only minimally affected by quizartinib and midostaurin (53), and that the co-existence of wild-type FLT3 can dampen the efficacy of FLT3 inhibitors in FLT3-mutated AML cells in vitro and in vivo (54). As a result, signaling through wild-type FLT3 in MOLM-13 cells could contribute to resistance mechanisms not observed in FLT3-ITD^+/+^ AML cells. However, most drug combinations that demonstrated synergistic effects in MOLM-13 cells also showed synergy in the homozygous FLT3-ITD^+/+^ MV4-11 cell line, as well as in FLT3-ITD-mutated primary AML patient samples with a high FLT3-ITD variant allele frequency, indicative of a homozygous mutation. These findings suggest that the drug combinations are effective in both genetic backgrounds and that the underlying primed resistance mechanisms are not unique to the MOLM-13 cell line.

We observed an up-regulation of GSPT1 in both midostaurin and quizartinib pre-resistance signatures. GSPT1 degraders have emerged as promising candidates for novel AML therapies, showing efficacy across various genetic backgrounds (33,55). The CD33-targeting antibody-drug conjugate BMS-986497, which delivers the GSPT1 degrader SMol006 specifically to CD33-expressing cells, is currently under clinical investigation for relapsed or refractory AML—both as monotherapy and in combination with azacitidine and venetoclax (ClinicalTrilas.gov: NCT06419634). This trial follows the early termination of clinical studies involving the GSPT1 degrader CC-90009 (ClinicalTrials.gov: NCT02848001 and NCT04336982), reportedly due to an unfavorable change in the risk-benefit profile. Mechanistically, pharmacological degradation of GSPT1 leads to impaired translation termination, activation of the integrated stress response pathway, and TP53-independent cell death in AML cells, with minimal effect on normal hematopoietic stem cells (56). Further, GSPT1 depletion attenuates translation initiation and cell cycle progression by inhibiting mTOR signaling downstream of AKT activation, independent of the Erk1/2 pathway (57). Whether increased GSPT1 expression directly up-regulates mTOR signaling and partly explains how GSPT1 could confer resistance against FLT3 inhibitors, warrants further investigation. Interestingly, hyperactivation of the mTOR signaling pathway has been found to attenuate the response to CC-90009 by blocking GSPT1 degradation (58). Therefore, the synergistic effect of CC-90009 and quizartinib or midostaurin may partly stem from FLT3 inhibitors reducing downstream mTOR signaling, thus enhancing the efficacy of CC-90009 in FLT3-ITD-positive AML cells. Importantly, we also observed enhanced sensitivity to quizartinib following CRISPR-Cas9-mediated GSPT1 knockout, even when GSPT1 was depletion was incomplete, underscoring its role in mediating resistance to FLT3 inhibition. Moreover, while quizartinib alone was largely ineffective in primary AML patient samples at clinically relevant concentrations (peak serum concentration range from 250 nM during induction therapy to 945 nM at maintenance therapy with VANFLYTA^®^; https://www.accessdata.fda.gov/drugsatfda_docs/label/2023/216993s000lbl.pdf), its combination with low doses of CC-90009 led to strong inhibitory effects across all samples. In addition, in an AML PDX model, CC-90009 alone showed limited efficacy at the tested dose, but its combination with quizartinib significantly enhanced anti-leukemic effects and improved overall survival compared to quizartinib alone. Further studies are warranted to clarify the mechanism underlying the observed drug synergy and to assess effects on normal hematopoietic cells. In the PDX mouse model, apart from transient ruffled fur, no other treatment-related clinical signs were noted, indicating that the treatments were well tolerated. Moreover, no evidence of clinical signs (paleness or bruising) of impaired hematopoiesis (anemia or thrombocytopenia) were observed. Together, these findings highlight the therapeutic potential of combining GSPT1 degraders with FLT3 inhibitors to enhance treatment efficacy while reducing the toxicities and side-effects associated with high-dose FLT3 inhibitor treatments.

In summary, ReSisTrace lineage-tracing offers a powerful tool for elucidating intricate cellular states that prime treatment resistance also in hematological cancers, as demonstrated here in the context of FLT3-ITD-mutated AML. Furthermore, our study unveils novel synergistic and pre-sensitizing drugs that could potentially be used to increase FLT3 inhibitor treatment efficacy and prevent emergence of treatment resistance against FLT3 inhibitors in FLT3-ITD-positive AML.

## Supplementary Material

Fig. S1

Fig. S2

Fig. S3

Fig. S4

Fig. S5

Fig. S6

Fig. S7

Table S1

Table S2

Table S3

Table S4

Table S5

Table S6

Table S7

Table S8

Table S9

## Data Availability

The ReSisTrace scRNA-seq raw data are publicly accessible through the NCBI Gene Expression Omnibus (GEO, RRID:SCR_005012) under accession number GSE306484. The L1000 data analyzed in this study were obtained from a previously published work (26); the corresponding raw data are available in GEO under accession numbers GSE92742 and GSE70138. All additional raw data generated during this study are available upon request from the corresponding author.

## Statement of significance

Single-cell lineage tracing with ReSisTrace reveals cellular states that prime treatment resistance and identifies approaches to sensitize FLT3-ITD-positive acute myeloid leukemia to FLT3 inhibitors.

## Code availability

The source code for data analysis is available at GitHub (https://github.com/TangSoftwareLab/ReSisTrace_AML).

## Abbreviations

AML: acute myeloid leukemia
DTP: drug-tolerant persister
FBS: fetal bovine serum
gRNA: guide RNA
GSEA: Gene Set Enrichment Analysis
HSA: Highest Single Agent
ITD: internal tandem duplication
MCM: Mononuclear Cell Medium
MRD: measurable residual disease
PDX: patient-derived xenograft
scRNA-seq: single-cell RNA-sequencing
TKD: tyrosine kinase domain
ZIP: Zero Interaction Potency

## References

[1] Döhner H, Weisdorf DJ, Bloomfield CD (2015). Acute Myeloid Leukemia. N Engl J Med.

[2] Kazi JU, Rönnstrand L (2019). FMS-like Tyrosine Kinase 3/FLT3: From Basic Science to Clinical
Implications. Physiol Rev.

[3] Yamamoto Y, Kiyoi H, Nakano Y, Suzuki R, Kodera Y, Miyawaki S (2001). Activating mutation of D835 within the activation loop of FLT3 in
human hematologic malignancies. Blood.

[4] Schnittger S, Schoch C, Dugas M, Kern W, Staib P, Wuchter C (2002). Analysis of FLT3 length mutations in 1003 patients with acute
myeloid leukemia: correlation to cytogenetics, FAB subtype, and prognosis in
the AMLCG study and usefulness as a marker for the detection of minimal
residual disease. Blood.

[5] Sheikhha MH, Awan A, Tobal K, Liu Yin JA (2003). Prognostic significance of FLT3 ITD and D835 mutations in AML
patients. Hematol J.

[6] Levis M (2017). Midostaurin approved for FLT3-mutated AML. Blood.

[7] Pulte ED, Norsworthy KJ, Wang Y, Xu Q, Qosa H, Gudi R (2021). FDA Approval Summary: Gilteritinib for Relapsed or Refractory
Acute Myeloid Leukemia with a FLT3 Mutation. Clin Cancer Res.

[8] Rummelt C, Gorantla SP, Meggendorfer M, Charlet A, Endres C, Döhner K (2021). Activating JAK-mutations confer resistance to FLT3 kinase
inhibitors in FLT3-ITD positive AML in vitro and in vivo. Leukemia.

[9] McMahon CM, Ferng T, Canaani J, Wang ES, Morrissette JJD, Eastburn DJ (2019). Clonal Selection with RAS Pathway Activation Mediates Secondary
Clinical Resistance to Selective FLT3 Inhibition in Acute Myeloid
Leukemia. Cancer Discov.

[10] Alotaibi AS, Yilmaz M, Kanagal-Shamanna R, Loghavi S, Kadia TM, DiNardo CD (2021). Patterns of Resistance Differ in Patients with Acute Myeloid
Leukemia Treated with Type I versus Type II FLT3 inhibitors. Blood Cancer Discov.

[11] Smith CC, Levis MJ, Perl AE, Hill JE, Rosales M, Bahceci E (2022). Molecular profile of FLT3-mutated relapsed/refractory patients
with AML in the phase 3 ADMIRAL study of gilteritinib. Blood Adv.

[12] Bell CC, Fennell KA, Chan YC, Rambow F, Yeung MM, Vassiliadis D (2019). Targeting enhancer switching overcomes non-genetic drug
resistance in acute myeloid leukaemia. Nat Commun.

[13] Petti AA, Khan SM, Xu Z, Helton N, Fronick CC, Fulton R (2022). Genetic and Transcriptional Contributions to Relapse in Normal
Karyotype Acute Myeloid Leukemia. Blood Cancer Discov.

[14] Shaffer SM, Dunagin MC, Torborg SR, Torre EA, Emert B, Krepler C (2017). Rare cell variability and drug-induced reprogramming as a mode of
cancer drug resistance. Nature.

[15] Cotton JL, Estrada Diez J, Sagar V, Chen J, Piquet M, Alford J (2023). Expressed Barcoding Enables High-Resolution Tracking of the
Evolution of Drug Tolerance. Cancer Res.

[16] Pellecchia S, Franchini M, Viscido G, Arnese R, Gambardella G (2024). Single cell lineage tracing reveals clonal dynamics of anti-EGFR
therapy resistance in triple negative breast cancer. Genome Med.

[17] Ramirez M, Rajaram S, Steininger RJ, Osipchuk D, Roth MA, Morinishi LS (2016). Diverse drug-resistance mechanisms can emerge from drug-tolerant
cancer persister cells. Nat Commun.

[18] Dai J, Zheng S, Falco MM, Bao J, Eriksson J, Pikkusaari S (2024). Tracing back primed resistance in cancer via sister
cells. Nat Commun.

[19] Subramanian A, Narayan R, Corsello SM, Peck DD, Natoli TE, Lu X (2017). A Next Generation Connectivity Map: L1000 Platform and the First
1,000,000 Profiles. Cell.

[20] Zheng GX, Terry JM, Belgrader P, Ryvkin P, Bent ZW, Wilson R (2017). Massively parallel digital transcriptional profiling of single
cells. Nat Commun.

[21] Smith T, Heger A, Sudbery I (2017). UMI-tools: modeling sequencing errors in Unique Molecular
Identifiers to improve quantification accuracy. Genome Res.

[22] Hao Y, Hao S, Andersen-Nissen E, Mauck WM, Zheng S, Butler A (2021). Integrated analysis of multimodal single-cell
data. Cell.

[23] Liberzon A, Subramanian A, Pinchback R, Thorvaldsdóttir H, Tamayo P, Mesirov JP (2011). Molecular signatures database (MSigDB) 3.0. Bioinformatics.

[24] Subramanian A, Tamayo P, Mootha VK, Mukherjee S, Ebert BL, Gillette MA (2005). Gene set enrichment analysis: a knowledge-based approach for
interpreting genome-wide expression profiles. Proc Natl Acad Sci U S A.

[25] Lamb J, Crawford ED, Peck D, Modell JW, Blat IC, Wrobel MJ (2006). The Connectivity Map: using gene-expression signatures to connect
small molecules, genes, and disease. Science.

[26] Douglass EF, Allaway RJ, Szalai B, Wang W, Tian T, Fernández-Torras A (2022). A community challenge for a pancancer drug mechanism of action
inference from perturbational profile data. Cell Rep Med.

[27] Szalai B, Subramanian V, Holland CH, Alföldi R, Puskás LG, Saez-Rodriguez J (2019). Signatures of cell death and proliferation in perturbation
transcriptomics data-from confounding factor to effective
prediction. Nucleic Acids Res.

[28] Smirnov P, Safikhani Z, El-Hachem N, Wang D, She A, Olsen C (2016). PharmacoGx: an R package for analysis of large pharmacogenomic
datasets. Bioinformatics.

[29] Zheng S, Wang W, Aldahdooh J, Malyutina A, Shadbahr T, Tanoli Z (2022). SynergyFinder Plus: Toward Better Interpretation and Annotation
of Drug Combination Screening Datasets. Genomics Proteomics Bioinformatics.

[30] Eirew P, O’Flanagan C, Ting J, Salehi S, Brimhall J, Wang B (2022). Accurate determination of CRISPR-mediated gene fitness in
transplantable tumours. Nat Commun.

[31] Safont MM, Leitch C, Popa M, Gjerstad ME, Caulier B, Inderberg EM (2024). Protocol for the development of a bioluminescent AML-PDX mouse
model for the evaluation of CAR T cell therapy. STAR Protoc.

[32] Jiang J, Griffin JD (2010). Wnt/β-catenin Pathway Modulates the Sensitivity of the
Mutant FLT3 Receptor Kinase Inhibitors in a GSK-3β Dependent
Manner. Genes Cancer.

[33] Hansen JD, Correa M, Alexander M, Nagy M, Huang D, Sapienza J (2021). CC-90009: A Cereblon E3 Ligase Modulating Drug That Promotes
Selective Degradation of GSPT1 for the Treatment of Acute Myeloid
Leukemia. J Med Chem.

[34] Jung DW, Kim WH, Park SH, Lee J, Kim J, Su D (2013). A unique small molecule inhibitor of enolase clarifies its role
in fundamental biological processes. ACS Chem Biol.

[35] Eisfelder BJ, Saygin C, Wynne J, Colton MW, Fischietti M, Beauchamp EM (2021). OTS167 blocks FLT3 translation and synergizes with FLT3
inhibitors in FLT3 mutant acute myeloid leukemia. Blood Cancer J.

[36] Karjalainen R, Pemovska T, Popa M, Liu M, Javarappa KK, Majumder MM (2017). JAK1/2 and BCL2 inhibitors synergize to counteract bone marrow
stromal cell-induced protection of AML. Blood.

[37] Piloto O, Wright M, Brown P, Kim KT, Levis M, Small D (2007). Prolonged exposure to FLT3 inhibitors leads to resistance via
activation of parallel signaling pathways. Blood.

[38] Lindblad O, Cordero E, Puissant A, Macaulay L, Ramos A, Kabir NN (2016). Aberrant activation of the PI3K/mTOR pathway promotes resistance
to sorafenib in AML. Oncogene.

[39] Joshi SK, Nechiporuk T, Bottomly D, Piehowski PD, Reisz JA, Pittsenbarger J (2021). The AML microenvironment catalyzes a stepwise evolution to
gilteritinib resistance. Cancer Cell.

[40] Traer E, Martinez J, Javidi-Sharifi N, Agarwal A, Dunlap J, English I (2016). FGF2 from Marrow Microenvironment Promotes Resistance to FLT3
Inhibitors in Acute Myeloid Leukemia. Cancer Res.

[41] Park HJ, Gregory MA, Zaberezhnyy V, Goodspeed A, Jordan CT, Kieft JS (2022). Therapeutic resistance in acute myeloid leukemia cells is
mediated by a novel ATM/mTOR pathway regulating oxidative
phosphorylation. Elife.

[42] Darici S, Zavatti M, Braglia L, Accordi B, Serafin V, Horne GA (2021). Synergistic cytotoxicity of dual PI3K/mTOR and FLT3 inhibition in
FLT3-ITD AML cells. Adv Biol Regul.

[43] Mulvihill MJ, Cooke A, Rosenfeld-Franklin M, Buck E, Foreman K, Landfair D (2009). Discovery of OSI-906: a selective and orally efficacious dual
inhibitor of the IGF-1 receptor and insulin receptor. Future Med Chem.

[44] Hopkins BD, Goncalves MD, Cantley LC (2020). Insulin-PI3K signalling: an evolutionarily insulated metabolic
driver of cancer. Nat Rev Endocrinol.

[45] Tegethoff J, Bischoff R, Saleh S, Blagojevic B, Merz KH, Cheng X (2017). Methylisoindigo and Its Bromo-Derivatives Are Selective Tyrosine
Kinase Inhibitors, Repressing Cellular Stat3 Activity, and Target CD133+
Cancer Stem Cells in PDAC. Molecules.

[46] Okamoto M, Hayakawa F, Miyata Y, Watamoto K, Emi N, Abe A (2007). Lyn is an important component of the signal transduction pathway
specific to FLT3/ITD and can be a therapeutic target in the treatment of AML
with FLT3/ITD. Leukemia.

[47] Voisset E, Lopez S, Chaix A, Georges C, Hanssens K, Prébet T (2010). FES kinases are required for oncogenic FLT3
signaling. Leukemia.

[48] Xiao Z, Hao Y, Liu B, Qian L (2002). Indirubin and meisoindigo in the treatment of chronic myelogenous
leukemia in China. Leuk Lymphoma.

[49] Lee CC, Lin CP, Lee YL, Wang GC, Cheng YC, Liu HE (2010). Meisoindigo is a promising agent with in vitro and in vivo
activity against human acute myeloid leukemia. Leuk Lymphoma.

[50] Basu B, Dean E, Puglisi M, Greystoke A, Ong M, Burke W (2015). First-in-Human Pharmacokinetic and Pharmacodynamic Study of the
Dual m-TORC 1/2 Inhibitor AZD2014. Clin Cancer Res.

[51] Jones RL, Kim ES, Nava-Parada P, Alam S, Johnson FM, Stephens AW (2015). Phase I study of intermittent oral dosing of the insulin-like
growth factor-1 and insulin receptors inhibitor OSI-906 in patients with
advanced solid tumors. Clin Cancer Res.

[52] Xiao Z, Qian L, Liu B, Hao Y (2000). Meisoindigo for the treatment of chronic myelogenous
leukaemia. Br J Haematol.

[53] Kawase T, Nakazawa T, Eguchi T, Tsuzuki H, Ueno Y, Amano Y (2019). Effect of Fms-like tyrosine kinase 3 (FLT3) ligand (FL) on
antitumor activity of gilteritinib, a FLT3 inhibitor, in mice xenografted
with FL-overexpressing cells. Oncotarget.

[54] Chen F, Ishikawa Y, Akashi A, Naoe T, Kiyoi H (2016). Co-expression of wild-type FLT3 attenuates the inhibitory effect
of FLT3 inhibitor on FLT3 mutated leukemia cells. Oncotarget.

[55] Perzolli A, Steinebach C, Krönke J, Gütschow M, Zwaan CM, Barneh F (2025). PROTAC-Mediated GSPT1 Degradation Impairs the Expression of
Fusion Genes in Acute Myeloid Leukemia. Cancers (Basel).

[56] Sellar RS, Sperling AS, Słabicki M, Gasser JA, McConkey ME, Donovan KA (2022). Degradation of GSPT1 causes TP53-independent cell death in
leukemia while sparing normal hematopoietic stem cells. J Clin Invest.

[57] Chauvin C, Salhi S, Jean-Jean O (2007). Human eukaryotic release factor 3a depletion causes cell cycle
arrest at G1 phase through inhibition of the mTOR pathway. Mol Cell Biol.

[58] Surka C, Jin L, Mbong N, Lu CC, Jang IS, Rychak E (2021). CC-90009, a novel cereblon E3 ligase modulator, targets acute
myeloid leukemia blasts and leukemia stem cells. Blood.

