# Supplementary material for: Single-Cell Lineage Tracing Uncovers Resistance Signatures and Sensitizing Strategies to FLT3 Inhibitors in Acute Myeloid Leukemia
Source: Cancer Res. Author manuscript; Available in PMC 2025 Dec 10. (PMC7618455; doi:10.1158/0008-5472.CAN-24-3753)
Supplement: Fig. S1 [file EMS211203-supplement-Fig__S1.pdf]

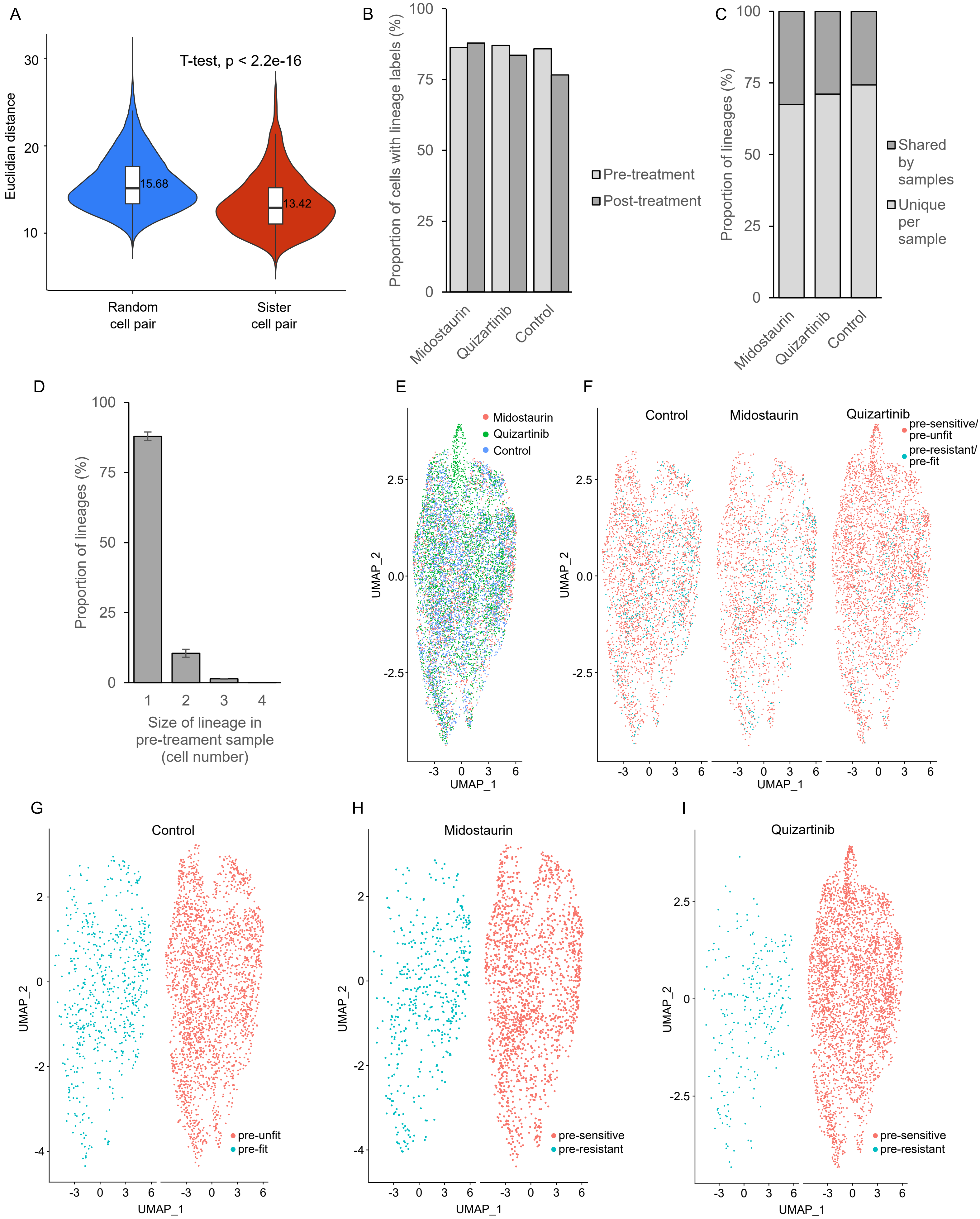

Fig. S1. Lineage labeling efficiency, label detection assessment, and sister cell similarity.
(A) Euclidean distance of sister cell pairs in comparison to random cell pairs in the pre-treatment samples ($P < 2.2e-16$, two-tailed t-test). (B) Proportion of cells with lineage labels detected per sample. (C) Proportion of unique and shared barcodes in each pre-treatment sample. (D) Average proportion of lineages with indicated numbers of cells within a pre-treatment sample. (E) UMAP projection of the pre-treatment samples. (F-G) UMAP projections of pre-resistant and pre-sensitive in each pre-treatment sample or pre-fit and pre-unfit cells in the DMSO-treated control.
