# Supplementary material for: Single-Cell Lineage Tracing Uncovers Resistance Signatures and Sensitizing Strategies to FLT3 Inhibitors in Acute Myeloid Leukemia
Source: Cancer Res. Author manuscript; Available in PMC 2025 Dec 10. (PMC7618455; doi:10.1158/0008-5472.CAN-24-3753)
Supplement: Fig. S2 [file EMS211203-supplement-Fig__S2.pdf]

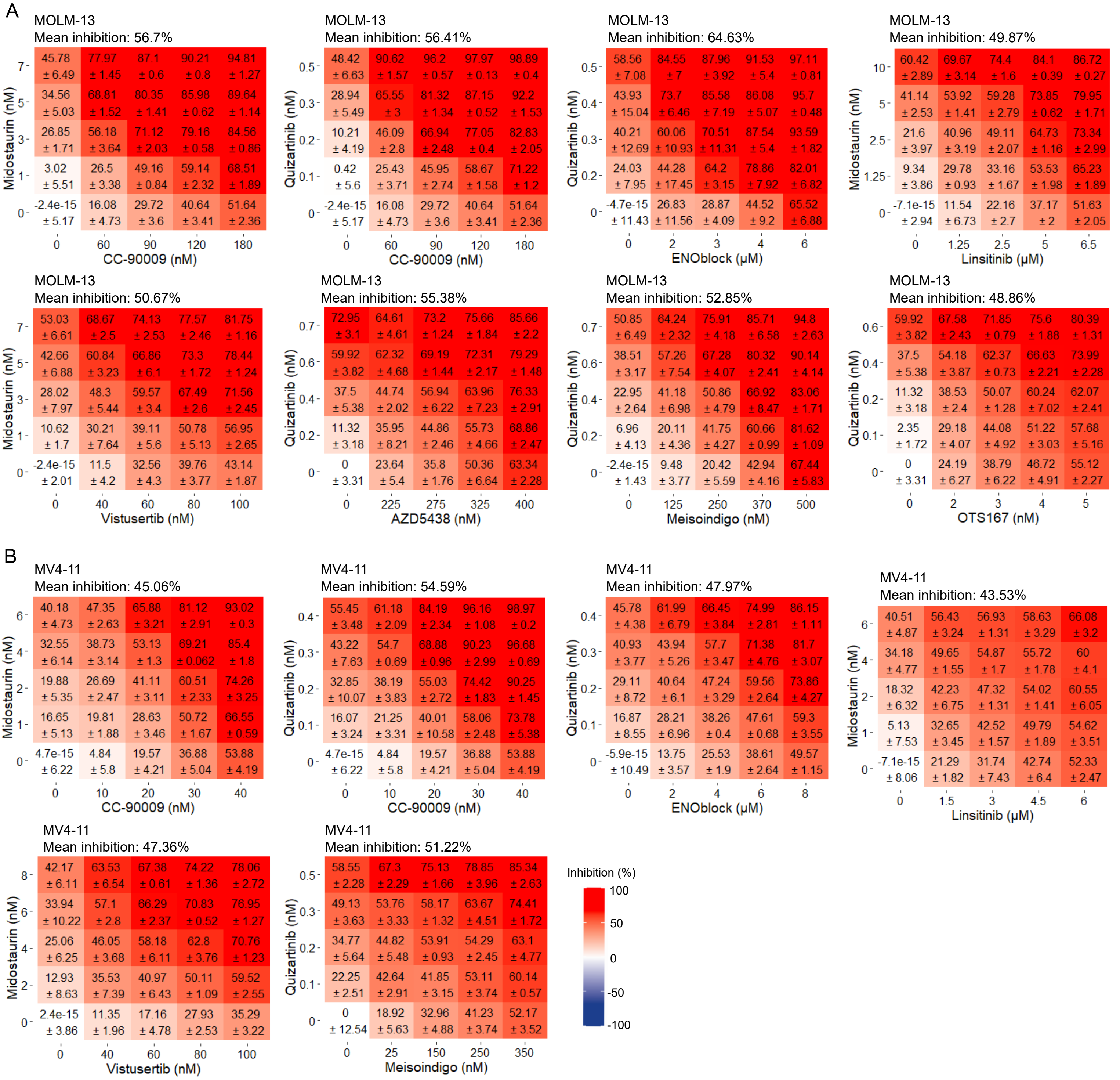

Fig. S2. Dose-response matrices for drug combinations in MOLM-13 and MV4-11 cell lines.

Dose-responses (mean ± standard deviation) for indicated drugs in combination with midostaurin or quizartinib in MOLM-13 (A) and MV4-11 (B) cells. Cells were first treated with the drug on horizontal axis with indicated concentrations, and after 24 hours, midostaurin or quizartinib were added for 72 hours with concentrations indicated in the vertical axis.
