# Supplementary material for: Single-Cell Lineage Tracing Uncovers Resistance Signatures and Sensitizing Strategies to FLT3 Inhibitors in Acute Myeloid Leukemia
Source: Cancer Res. Author manuscript; Available in PMC 2025 Dec 10. (PMC7618455; doi:10.1158/0008-5472.CAN-24-3753)
Supplement: Fig. S3 [file EMS211203-supplement-Fig__S3.pdf]

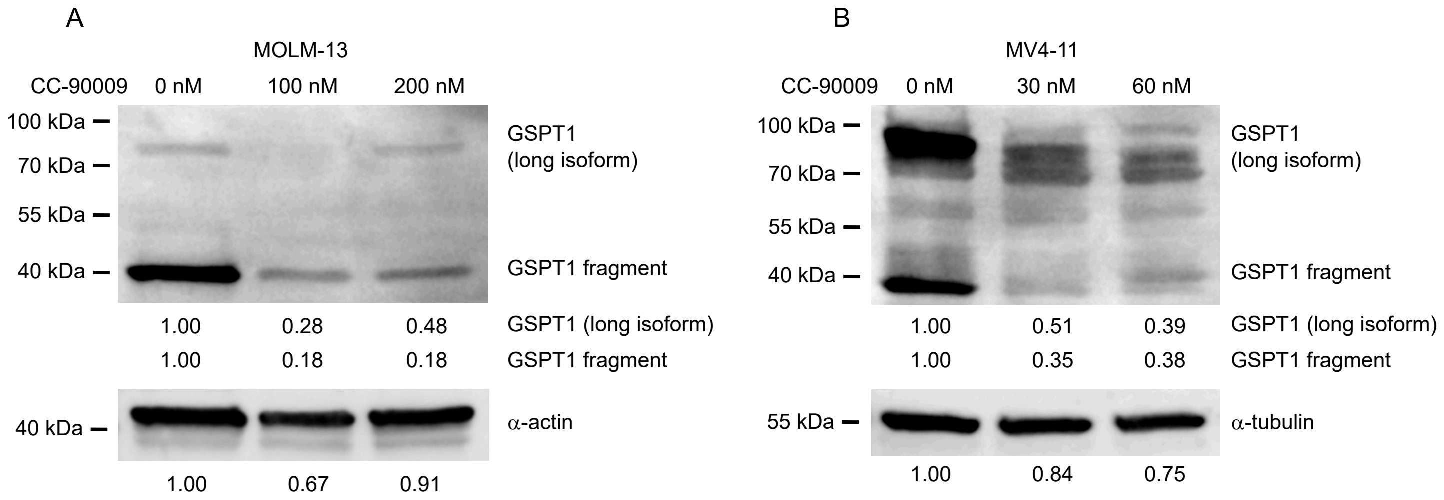

Fig. S3. GSPT1 protein levels after CC-90009 treatment.
Western blot analysis of GSPT1 protein levels in MOLM-13 (A) and MV4 -11 (B) cell lines after treating the cells with DMSO or indicated concentrations of CC-90009 for 24 hours. Alpha-actin or alpha-tubulin were used as loading controls. Relative levels of each protein or protein fragment compared to the DMSO control are shown below.
