# Supplementary material for: Single-Cell Lineage Tracing Uncovers Resistance Signatures and Sensitizing Strategies to FLT3 Inhibitors in Acute Myeloid Leukemia
Source: Cancer Res. Author manuscript; Available in PMC 2025 Dec 10. (PMC7618455; doi:10.1158/0008-5472.CAN-24-3753)
Supplement: Fig. S4 [file EMS211203-supplement-Fig__S4.pdf]

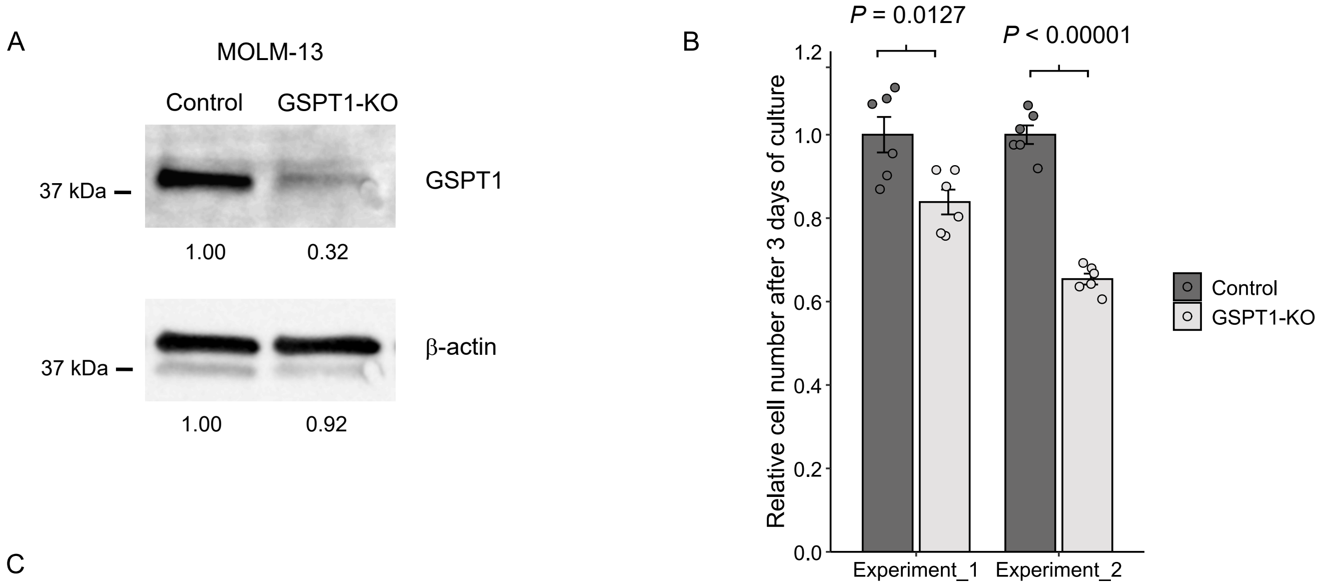

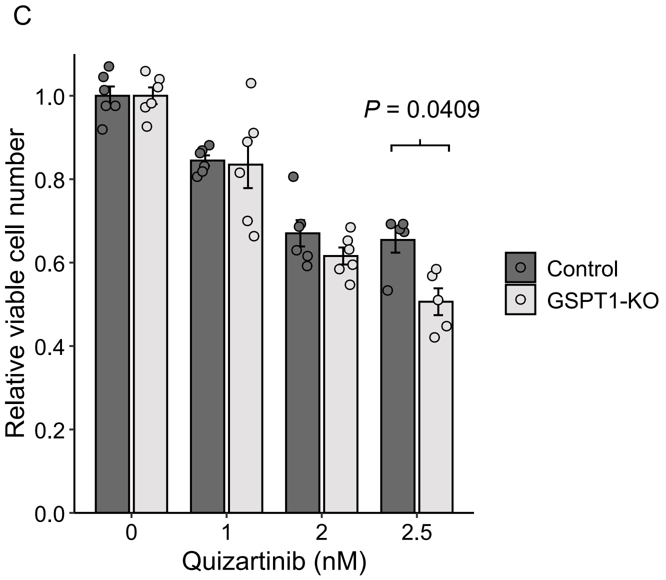

Fig. S4. Effect of GSPT1 knockout on FLT3 inhibitor sensitivity. (A) Western blot analysis of GSPT1 protein levels in MOLM-13 control and GSPT1-KO pool cells. Beta-actin was used as a loading control. (B) Relative MOLM-13 control and GSPT1-KO cell numbers after 3 days of culture in two replicate experiments. Experiments were started after puromycin selection on day 8 (Experiment_1) or day 7 (Experiment_2) after transduction. Data were normalized to the mean cell number of the control cells. (C) Relative viable MOLM-13 control and GSPT1-KO cell numbers after quizartinib treatment for 72 hours. Data were normalized to the mean viable cell number of the DMSO-treated (0 nM) cells. (B and C) Data were plotted as individual measurements and mean ± standard error of mean. Statistical significance was determined by two-tailed t-test and *P*-values were adjusted by Holm's method.
