# Supplementary material for: Single-Cell Lineage Tracing Uncovers Resistance Signatures and Sensitizing Strategies to FLT3 Inhibitors in Acute Myeloid Leukemia
Source: Cancer Res. Author manuscript; Available in PMC 2025 Dec 10. (PMC7618455; doi:10.1158/0008-5472.CAN-24-3753)
Supplement: Fig. S5 [file EMS211203-supplement-Fig__S5.pdf]

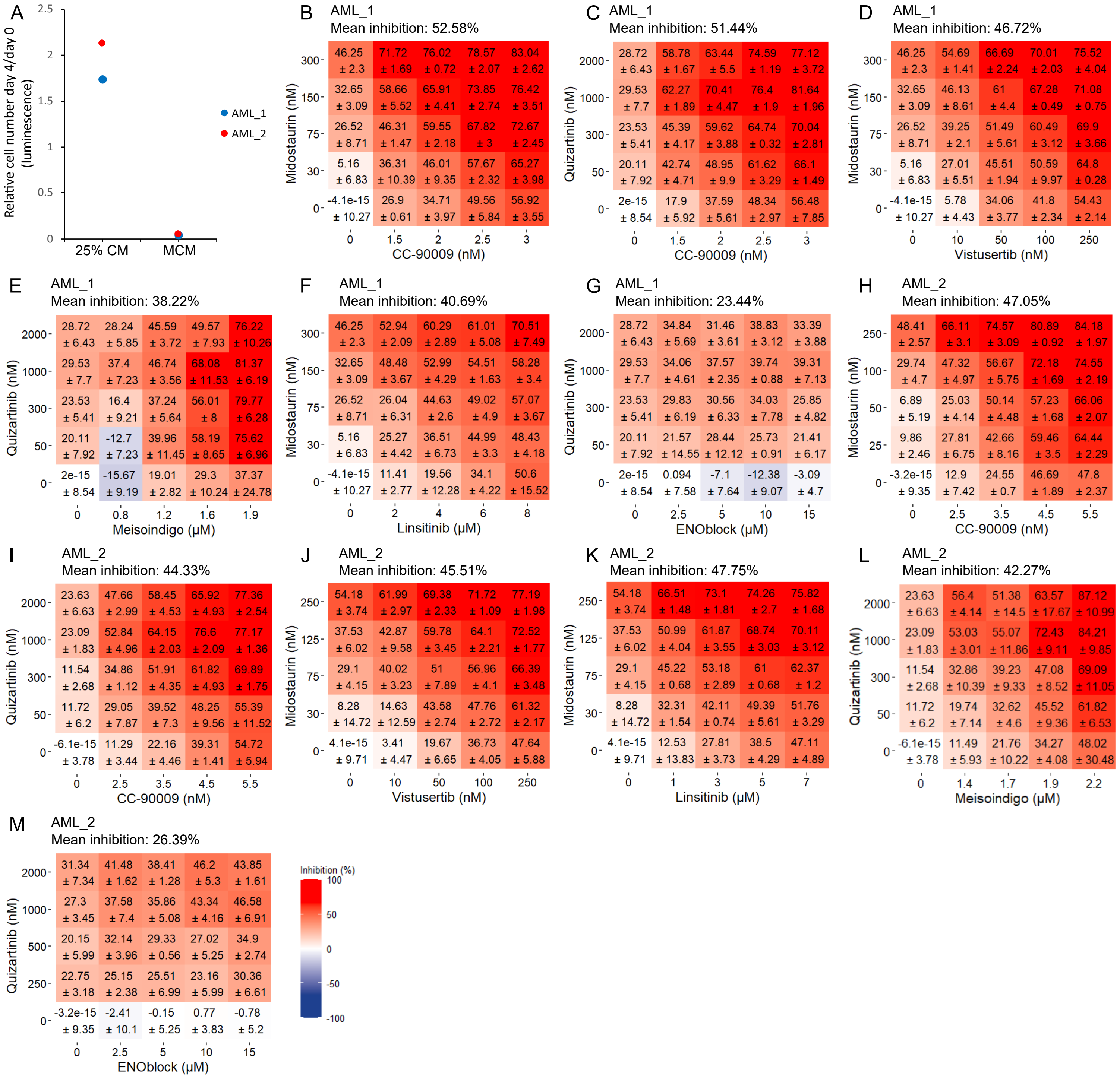

Fig. S5. Drug combination testing in primary AML patient samples.

(A) Relative cell number (CTG luminescence signal) in two FLT3-ITD-mutated AML patient samples after 4 days of culture in 25% HS-5 cell-conditioned medium (CM) or Mononuclear Cell Medium (MCM). (B-M) Dose-responses (mean inhibition % ± standard deviation) for indicated drugs in combination with midostaurin or quizartinib in patient sample AML_1 (B-G) and AML_2 (H-M). Cells were first treated with the drug on horizontal axis with indicated concentrations, and after 24 hours midostaurin or quizartinib were added for 72 hours with concentrations indicated in the vertical axis.
