# Supplementary material for: Single-Cell Lineage Tracing Uncovers Resistance Signatures and Sensitizing Strategies to FLT3 Inhibitors in Acute Myeloid Leukemia
Source: Cancer Res. Author manuscript; Available in PMC 2025 Dec 10. (PMC7618455; doi:10.1158/0008-5472.CAN-24-3753)
Supplement: Fig. S6 [file EMS211203-supplement-Fig__S6.pdf]

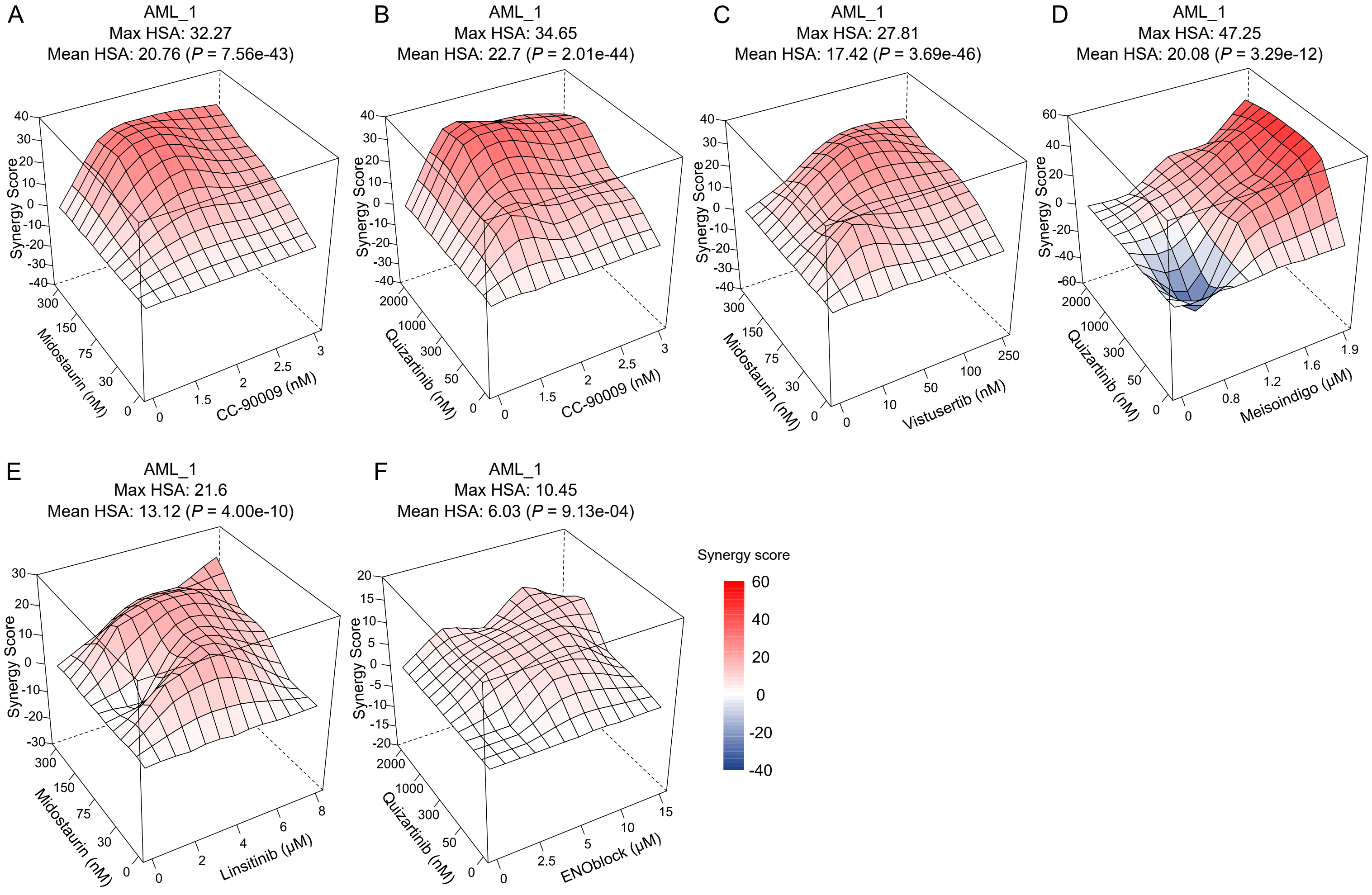

Fig. S6. Synergistic effects of drug combinations in primary AML patient samples.

(A-F) HSA synergy landscapes for the indicated drugs in combination with midostaurin or quizartinib in the primary AML patient sample AML_1. *P*-values were determined by bootstrapping test.
