# Supplementary material for: Single-Cell Lineage Tracing Uncovers Resistance Signatures and Sensitizing Strategies to FLT3 Inhibitors in Acute Myeloid Leukemia
Source: Cancer Res. Author manuscript; Available in PMC 2025 Dec 10. (PMC7618455; doi:10.1158/0008-5472.CAN-24-3753)
Supplement: Fig. S7 [file EMS211203-supplement-Fig__S7.pdf]

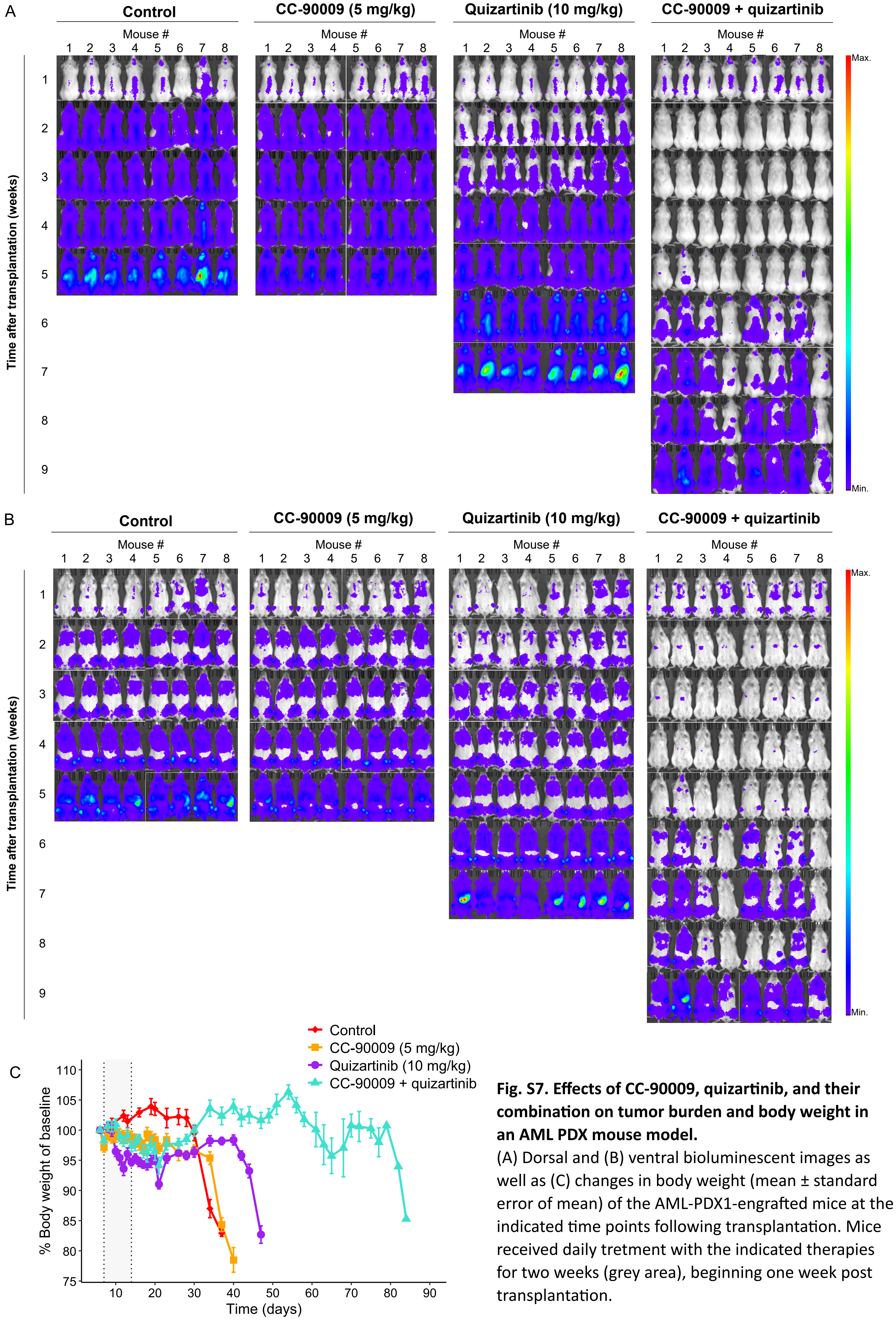

Fig. S7. Effects of CC-90009, quizartinib, and their combination on tumor burden and body weight in an AML PDX mouse model.
(A) Dorsal and (B) ventral bioluminescent images as well as (C) changes in body weight (mean ± standard error of mean) of the AML-PDX1-engrafted mice at the indicated time points following transplantation. Mice received daily tretment with the indicated therapies for two weeks (grey area), beginning one week post transplantation.
